# Integrative bulk and single-cell transcriptomic analysis identifies a migrasome-associated lncRNA signature predictive of prognosis and immune landscape in clear cell renal cell carcinoma

**DOI:** 10.3389/fimmu.2025.1638792

**Published:** 2025-08-20

**Authors:** Junlin Shen, Chun Wang, Mingpeng Zhang, Bin Chen, Liwei Liu, Jing Tian, Zhiqun Shang

**Affiliations:** Department of Urology, Tianjin Institute of Urology, The Second Hospital of Tianjin Medical University, Tianjin, China

**Keywords:** clear cell renal cell carcinoma, migrasome, prognostic signature, tumor mutation burden, tumor immune microenvironment, drug sensitivity, single-cell RNA sequencing

## Abstract

**Introduction:**

Clear cell renal cell carcinoma (ccRCC) is characterized by high recurrence and metastasis rates, leading to poor prognosis. Migrasomes, a class of organelles mediating intercellular communication, and long noncoding RNAs (lncRNAs) both play critical roles in tumor progression; however, the prognostic significance of migrasome-associated lncRNAs in ccRCC remains unclear.

**Methods:**

Migrasome-associated lncRNAs were identified using The Cancer Genome Atlas (TCGA) dataset, and a prognostic risk signature was constructed. The associations between the model and overall survival (OS), functional enrichment, tumor mutation burden (TMB), tumor microenvironment (TME) characteristics, immune evasion, and drug sensitivity were evaluated. Single-cell transcriptomic analysis was performed to determine cell type–specific expression patterns and intercellular communication networks. Functional roles of key lncRNAs were validated in vitro using qRT-PCR, CCK-8 proliferation assays, wound-healing assays, Transwell assays, colony formation assays, immunofluorescence, and Western blotting.

**Results:**

The risk signature stratified patients into high- and low-risk groups with significantly different survival outcomes. High-risk patients exhibited elevated TMB and enhanced immune evasion potential. Drug sensitivity analysis revealed distinct therapeutic response profiles between the groups. Single-cell transcriptomic analysis uncovered pronounced cellular heterogeneity and TME characteristics associated with the prognostic signature. High-risk cells were predominantly enriched within tumor epithelial clusters and displayed distinct intercellular communication patterns. Knockdown of FOXD2-AS1 markedly suppressed tumor cell proliferation and migration and reduced the expression of migrasome marker proteins.

**Discussion:**

This study presents a novel migrasome-associated lncRNA risk signature with significant prognostic and therapeutic implications for ccRCC. The signature captures distinct immune, genomic, and pharmacologic features, and its core lncRNAs may promote tumor progression through migrasome-mediated signaling pathways, warranting further mechanistic investigation.

## 1 Introduction

Renal cell carcinoma (RCC) ranks among the most prevalent kidney malignancies, with clear cell renal cell carcinoma (ccRCC) representing the predominant subtype, accounting for the majority of RCC cases (1). Despite recent advances in diagnostic and therapeutic strategies, the prognosis of ccRCC remains dismal, particularly in advanced disease stages, due to the high propensity for metastasis and recurrence (2). As such, identifying effective prognostic biomarkers and developing stratification models for risk assessment have become urgent to tailor personalized treatments and improve patient outcomes. Recent research has introduced migrasomes as a newly identified class of cellular organelles produced during cell migration (3). Migrasome formation occurs as cells leave retraction fibers during migration, with vesicles forming at fiber tips or intersections, requiring cell migration and actin polymerization. These vesicles transport cytosolic contents, facilitating intercellular communication (3). Migrasomes have been increasingly recognized for their roles in intercellular communication and as carriers of proteins, RNAs, and other bioactive molecules (4). Functionally, migrasomes play key roles in various physiological and pathological processes, including critical signaling pathways in embryonic development and cancer metastasis (5, 6). Recent studies have shown that highly migratory glioblastoma cells are capable of generating migrasomes. Additionally, migrasomes are enriched with endoplasmic reticulum-associated proteins, and increased endoplasmic reticulum stress has been demonstrated to promote migrasome formation in these cells (7). In pancreatic cancer, migrasomes enriched with CXCL5 and TGF-β1 recruit immune cells and induce immunosuppressive, tumor-promoting phenotypes, thereby enhancing malignancy and facilitating immune evasion (8). In summary, migrasomes are organelles of substantial importance in revealing mechanisms of intercellular interactions. Recent studies emphasize their crucial regulatory roles across various physiological activities and disease progression, offering new insights into disease diagnosis and prognosis. The role of long non-coding RNAs (lncRNAs), which are transcripts over 200 nucleotides in length and do not encode proteins, is increasingly prominent in cancer biology (9). LncRNAs are involved in transcriptional and post-transcriptional regulation as well as epigenetic modulation of gene expression (10). These molecules participate in critical oncogenic and tumor-suppressive pathways, regulating cellular processes such as proliferation, apoptosis, and migration (11). In ccRCC, several lncRNAs have been identified as influential in cancer cell behavior, with some emerging as promising biomarkers for diagnosis, prognosis, and treatment response (12, 13). Importantly, while several lncRNAs are involved in RCC, the subset specifically associated with migrasome-related genes remains largely unstudied. Given the essential roles of both migrasomes and lncRNAs in cancer progression, there is an increasing interest in understanding migrasome-associated lncRNAs as potential mediators in ccRCC, particularly regarding their prognostic value.

In this study, we constructed a prognostic signature based on migrasome-related lncRNAs for ccRCC. By stratifying patients into high- and low-risk groups, this model aims to predict survival outcomes and assist in clinical decision-making. Our findings provide new insights into the role of migrasome-associated lncRNAs in ccRCC progression, highlighting their significance as prognostic biomarkers and potential targets for therapeutic intervention.

## 2 Methods

### 2.1 Acquisition of transcriptomic and clinical data for clear cell renal cell carcinoma from TCGA

Transcriptomic and clinical data for clear cell renal cell carcinoma (ccRCC) were retrieved from The Cancer Genome Atlas (TCGA) to enable an integrated analysis of gene expression and patient characteristics. A total of 614 RNA sequencing (RNA-seq) files from 533 cases were obtained, comprising 542 tumor samples and 72 normal samples. Additionally, clinical data files were available for 537 cases, resulting in 537 clinical data files. The Genomic Data Commons (GDC) Data Portal (https://portal.gdc.cancer.gov) was used to access these harmonized datasets, ensuring consistency and quality control. Our use of public databases fully complies with the relevant ethical guidelines and regulations.

### 2.2 Construction of a migrasome-related lncRNA risk prognostic signature

Data preprocessing and analysis were conducted using the *Limma* package to identify lncRNAs associated with migrasome-related genes. Correlations were filtered with an absolute correlation coefficient > 0.4 and p-value < 0.001 to select significant lncRNAs. Expression data for these lncRNAs were obtained from transcriptomic data. Visualization was performed using the “ggalluvial” package, where a Sankey diagram was constructed to illustrate lncRNAs with significant correlations to migrasome-related genes. The dataset was then randomly divided into a training set and a testing set to minimize selection bias. Consistent filtering criteria were applied throughout the survival analysis, and the model was validated in both datasets to enhance the robustness and reproducibility of the results. In the training set, univariate Cox regression analysis was conducted to identify lncRNAs significantly associated with overall survival, with a significance threshold of p < 0.001. These significant lncRNAs were subsequently subjected to Lasso regression analysis for further variable selection and to construct a Lasso regression model. Cross-validation was performed to determine the optimal λ value, resulting in a simplified model. This approach was used to determine the optimal regularization parameter (λ), allowing us to control model complexity and retain only the most informative variables, thereby effectively reducing the risk of overfitting.

Based on the lncRNAs selected by Lasso, a multivariate Cox regression model was constructed to develop a prognostic signature. The model was further refined and optimized using the stepwise selection method, resulting in a final prognostic signature.

### 2.3 Correlation analysis between migrasome-related genes and signature lncRNAs

To investigate the correlation between migrasome-related genes and the lncRNAs included in the prognostic signature, we performed a correlation analysis using gene expression data. Expression matrices were constructed for both migrasome genes and lncRNAs. For each gene-lncRNA pair, the Pearson correlation coefficient and corresponding p-value were calculated, with significance levels defined as ***p < 0.001, **p < 0.01, and *p < 0.05. Correlation values were visualized in a heatmap, with color intensity reflecting the strength of the correlation.

### 2.4 Survival analysis based on migrasome-related lncRNA signature

To assess survival outcomes based on risk stratification, we performed Kaplan-Meier survival analyses for overall survival (OS) and progression-free survival (PFS). For OS, survival curves were plotted for training test, and combined all sets, using risk status (high vs. low) as the grouping variable. PFS was evaluated using the clinical follow-up data. Statistical significance was determined using the log-rank test, with p-values adjusted as needed for clarity (p < 0.001).

### 2.5 Risk stratification analysis of signature lncRNAs

Risk stratification plots were created to display the distribution of risk scores, survival status, and expression heatmap for key signature lncRNAs across the training, testing, and combined all datasets. For each dataset, samples were ordered by increasing risk score and divided into high- and low-risk groups based on the median score. The risk score plot illustrates the distribution for both groups, with a dashed line indicating the threshold between high and low-risk classifications. In the survival status plot, patient survival times are shown, where red represents deceased status and green indicates alive status. Finally, a heatmap was generated to show the expression levels of signature lncRNAs across high- and low-risk groups, with values scaled by row and annotated by risk classification.

### 2.6 Independent prognostic analysis via cox regression

Univariate and multivariate Cox regression analyses were conducted to evaluate the independent prognostic value of clinical factors and the lncRNA-based risk score. In the univariate analysis, clinical factors such as age, grade, stage, and the lncRNA risk score were individually assessed using Cox proportional hazards models. Statistical significance was determined with a threshold of p < 0.05, and significant variables were included in the multivariate Cox regression model to control for potential confounding factors. The analysis results include the hazard ratio (HR), 95% confidence interval (CI), and corresponding p-value for each factor.

### 2.7 ROC analysis for prognostic performance

We conducted time-dependent ROC analysis to assess the migrasome-related lncRNA signature’s predictive accuracy at 1, 3, and 5 years for survival. AUC values were used to quantify the signature’s prognostic strength. To further evaluate its independence as a prognostic indicator, we compared the 5-year ROC of the signature against clinical factors (age, gender, stage and grade).

### 2.8 Prognostic evaluation and nomogram analysis

To assess the prognostic performance of the lncRNA-based risk signature, we calculated the time-dependent concordance index (C-index) using the *pec* and “survcomp” R packages, and compared it with clinical variables including age, gender, stage, and grade. Cox models were built via the “cph” function, and C-index values were estimated using 1,000 bootstrap iterations. A nomogram was constructed based on multivariate Cox regression to predict 1-, 3-, and 5-year overall survival, using the “regplot” package. The corresponding risk scores were computed and the calibration curves were generated with 1,000 bootstraps to evaluate consistency between predicted and actual survival probabilities.

### 2.9 PCA analysis

PCA analysis was performed on four gene expression datasets to evaluate the distribution of high- and low-risk patients. Expression data from each dataset (all genes, migrasome-related genes, migrasome-related lncRNAs, and risk lncRNAs from the prognostic signature) were loaded, filtered for low expression, and log-transformed. Normal samples were excluded based on TCGA sample identifiers. PCA was conducted using “prcomp” with scaling, and risk groups were visualized with “scatterplot3d”, with color coding to represent high- and low-risk classifications.

### 2.10 GO enrichment analysis and KEGG pathway enrichment analysis of differentially expressed genes

We performed GO enrichment analysis on differentially expressed genes, covering biological process (BP), cellular component (CC), and molecular function (MF) categories. Using the “enrichGO” function in the “clusterProfiler” package, we set significance thresholds at p < 0.05 and q < 0.05. Enrichment results were visualized with bar and circular plots, where the bar plot displayed the top 10 enriched GO terms in each category, and the circular plot illustrated gene proportions and enrichment significance across GO terms. KEGG pathway enrichment analysis was performed on differentially expressed genes using the “enrichKEGG” function from the “clusterProfiler” package, with p < 0.05 and q < 0.05 as thresholds for significance. The most enriched pathways were visualized in a bar plot, capturing key biological functions associated with the genes, particularly those involved in immune response, cell signaling, and tissue structural integrity.

### 2.11 GSEA analysis of high and low-risk groups

To investigate the functional and pathway enrichment between high- and low-risk groups, we performed Gene Set Enrichment Analysis (GSEA). After processing the gene expression data and calculating log fold changes, GSEA was conducted using KEGG pathway terms, with enrichment results filtered at a p-value threshold of 0.05. We visualized the top five enriched functional terms for each risk group, revealing significant differences in enriched functions and pathways between the high- and low-risk categories.

### 2.12 Acquisition of TCGA mutation data

We also downloaded TCGA mutation data through the TCGA GDC portal. The final mutation dataset contained comprehensive mutation information for the selected ccRCC cases, providing a solid basis for further mutational landscape and correlation analyses.

### 2.13 Analysis of tumor microenvironment

Tumor microenvironment (TME) scores, including StromalScore, ImmuneScore, and ESTIMATEScore, were compared between high- and low-risk groups using violin plots with overlaid boxplots. The Wilcoxon test assessed group differences, with significance levels displayed. Immune cell composition was analyzed using CIBERSORT to estimate relative abundances in high- and low-risk groups, filtering results with a p-value threshold of <0.05. Samples were matched to risk groups, and differences in immune composition were visualized through bar plots for overall abundances and box plots for statistically significant differences. To evaluate immune function differences between high- and low-risk groups, ssGSEA was applied to the expression matrix using curated immune-related gene sets. Low-expressed genes were filtered out, and immune function scores were computed for each sample. Distribution differences between risk groups were then visualized using box plots.

### 2.14 Analysis of tumor mutational burden

Tumor mutational burden (TMB) was calculated to assess the mutational landscape of clear cell renal cell carcinoma (ccRCC) cases. TMB was defined as the total number of somatic, coding, base substitution, and indel mutations per megabase (Mb) of the coding genome. TMB was analyzed by examining the top 15 genes with the highest mutation frequencies. The “oncoplot” function from the “maftools” package was used to visualize the mutation data for each group, displaying the gene mutation frequencies in both high-risk and low-risk cohorts. To evaluate the prognostic impact of TMB and its interaction with risk status, samples were categorized as high or low TMB. Combined with risk groups, four subgroups were created: H-TMB + high-risk, H-TMB + low-risk, L-TMB + high-risk, and L-TMB + low-risk. Survival curves were plotted with “ggsurvplot” from “survminer” package. TIDE scores were extracted and analyzed to compare immune evasion potential between high- and low-risk groups.

### 2.15 Drug sensitivity analysis

Drug sensitivity analysis compared response variations between high- and low-risk groups across multiple drugs. Drug responses were log-transformed and tested for significance using the Wilcoxon test, with only those drugs meeting a p-value threshold of <0.001 visualized. Sensitivity differences for each drug were depicted through boxplots, contrasting high- and low-risk groups.

### 2.16 Single-cell RNA sequencing data acquisition and processing

Single-cell RNA sequencing (scRNA-seq) data were obtained from two publicly available GEO datasets: GSE222703 (samples GSM6929206, GSM6929208, and GSM6929210) and GSE152938 (samples GSM4630028 and GSM4630029), comprising a total of five primary clear cell renal cell carcinoma (ccRCC) samples. The raw UMI count matrices in 10X format were processed using the “Seurat” package in R, involving standard workflows for quality control, doublet removal, normalization, integration, and downstream analyses. During initial quality control, cells were filtered out if they expressed fewer than 300 genes, had fewer than 1,000 total UMI counts, or exhibited over 10% mitochondrial gene content. “DoubletFinder” was used to identify and exclude potential doublets, assuming a doublet formation rate of approximately 7.5–8.0%. After preprocessing, Seurat objects from individual samples were merged and batch effects were corrected using Harmony. Dimensionality reduction was then performed by principal component analysis (PCA) followed by UMAP embedding based on the top 20 Harmony dimensions. Cell type annotation was carried out manually based on differentially expressed marker genes identified using the “FindAllMarkers” function. Clusters were annotated as CD4^+^ T cells, CD8^+^ T cells, tumor epithelial cells, inflammatory monocytes, tumor-associated macrophages (TAMs), natural killer (NK) cells, endothelial cells, cycling CD8^+^ T cells, proximal tubular epithelial cells, mural cells, mast cells, B cells, plasma cells, and plasmacytoid dendritic cells (pDCs). To assess the prognostic relevance of the lncRNA signature at the single-cell level, a risk score was calculated for each cell using a linear combination of the normalized expression values of the seven signature lncRNAs, weighted by their Cox regression coefficients. Cells with non-zero scores were stratified into high- and low-risk groups using the median value as the cutoff. Differences in cell type composition between risk groups were quantified and visualized using stacked bar plots. The expression patterns of individual signature genes across cell populations were visualized using “FeaturePlot” and “DotPlot”. To investigate intercellular communication, we applied the “CellChat” package. Cell–cell interaction probabilities were inferred from the normalized expression matrix and annotated cell identities. Enriched signaling pathways, including MHC class I, MHC class II, complement, collagen, TNF, and VEGF were identified and visualized using circle plots, chord diagrams, and heatmaps, highlighting differences in communication networks associated with risk stratification.

### 2.17 Cell culture and transfection

OS-RC-2 cells and 786-O, obtained from the Cell Bank of the Chinese Academy of Sciences (China), were cultured in RPMI 1640 medium (VivaCell, China) supplemented with 10% fetal bovine serum (FBS; ExCell Bio, China) at 37°C in a humidified incubator with 5% CO2. Our use of these commercially available cell lines complies with all relevant ethical regulations. Transient knockdown of factors were achieved using small interfering RNA (siRNA) designed and synthesized by JTSBIO Co. (China). Transfections were performed using Lipofectamine™ 3000 reagent (Invitrogen, USA) according to the manufacturer’s instructions. The sequence of the siRNA used for transfection is provided in **Supplementary Table S1**.

### 2.18 Quantitative real-time PCR

Total RNA was extracted from cells using Trizol reagent (Invitrogen, USA) following the manufacturer’s protocol. Reverse transcription was performed using a reverse transcription kit (Thermo Scientific, USA) to synthesize cDNA. Quantitative real-time PCR (qRT-PCR) was conducted using SYBR Green PCR Master Mix (Roche), ensuring accurate quantification of gene expression. The primers used for qRT-PCR are listed in the **Supplementary Table S1**.

### 2.19 CCK-8 assay

Cell proliferation was evaluated using the Cell Counting Kit-8 (CCK-8) assay. OS-RC-2 cells and 786-O cells were seeded into 96-well plates at a density of 1000 cells per well and maintained under standard culture conditions. At designated time points, 10 μL of CCK-8 reagent was added to each well, followed by incubation at 37°C for 1 hour. Absorbance at 450 nm was measured using an enzymatic calibrator to determine cell viability.

### 2.20 Wound-healing assay

OS-RC-2 cells and 786-O cells were plated in six-well plates and allowed to reach 90-100% confluence. To create a simulated wound, a sterile 200 μL pipette tip was drawn through the cell monolayer. Detached cells and debris were removed with two rinses of phosphate-buffered saline (PBS), after which the cells were maintained in serum-free medium. Baseline wound areas were imaged under an inverted microscope at 10x magnification. After 24 hours of incubation, additional images were taken to assess wound closure. The percentage of wound closure was calculated using ImageJ software for quantitative analysis.

### 2.21 Colony formation assay

OS-RC-2 cells and 786-O cells were plated in six-well plates at a density of 500 cells per well and maintained in complete RPMI 1640 medium containing 10% fetal bovine serum under standard culture conditions (37°C, 5% CO_2_). Cells were cultured for 14 days, with media refreshed as required, until distinct colonies became visible. Afterward, the cells were rinsed with phosphate-buffered saline (PBS) and fixed with 4% paraformaldehyde for 15 minutes at room temperature. Colonies were then stained using a crystal violet solution, washed thoroughly to remove residual dye, air-dried, and captured for further analysis and comparison.

### 2.22 Cell migration assay

Transwell migration assays were conducted to assess the migratory capacity of OS-RC-2 cells and 786-O cells using Transwell chambers (Corning Costar, USA). The lower chamber was filled with medium containing serum, while the upper chamber contained serum-free medium. A total of 10,000 cells were seeded into each upper chamber. After 48 hours of incubation, cells were washed with phosphate-buffered saline (PBS) and stained with crystal violet. Stained cells on the lower membrane were visualized under a microscope, and cell counts were quantified using ImageJ software.

### 2.23 Immunofluorescence staining

Cells grown on glass coverslips were fixed in 4% paraformaldehyde for 15 min, followed by permeabilization with 0.1% Triton X-100 for 10 min at room temperature. After blocking in 5% BSA for 1 h, cells were incubated with primary antibodies overnight at 4 °C. The next day, samples were washed with PBS and treated with fluorophore-conjugated secondary antibodies for 1 h in the dark at room temperature. Nuclei were stained with DAPI, and fluorescent images were acquired using a fluorescence microscope.

### 2.24 Western blot

Cells were placed in RIPA buffer containing PMSF (1:100), lysed on ice for 30 minutes, followed by ultrasonic lysis. Then, the mixture was centrifuged at 12,000×g for 20 minutes at 4°C, and the supernatants were collected. The protein concentration was determined using a BCA protein assay kit. Equal amounts of protein (20 μg per lane) were separated by 10% SDS-PAGE and then transferred onto PVDF membranes. The membranes were blocked with 5% non-fat milk in TBST for 1 hour at room temperature, after which they were incubated with primary antibodies against DNST1 (1:1000, 26203-1-AP, Proteintech, China), EOGT (1:1000, 27595-1-AP, Proteintech, China) and GAPDH (1:50000, 60004-1-Ig, Proteintech, China) at 4°C overnight. Subsequently, the membranes were washed three times with TBST and then incubated with secondary antibodies for 1 hour at room temperature. After an additional three washes with TBST, the protein bands were developed.

## 3 Results

### 3.1 Identification of prognostic migrasome-related lncRNAs and signature construction in ccRCC

**Figure 1:**
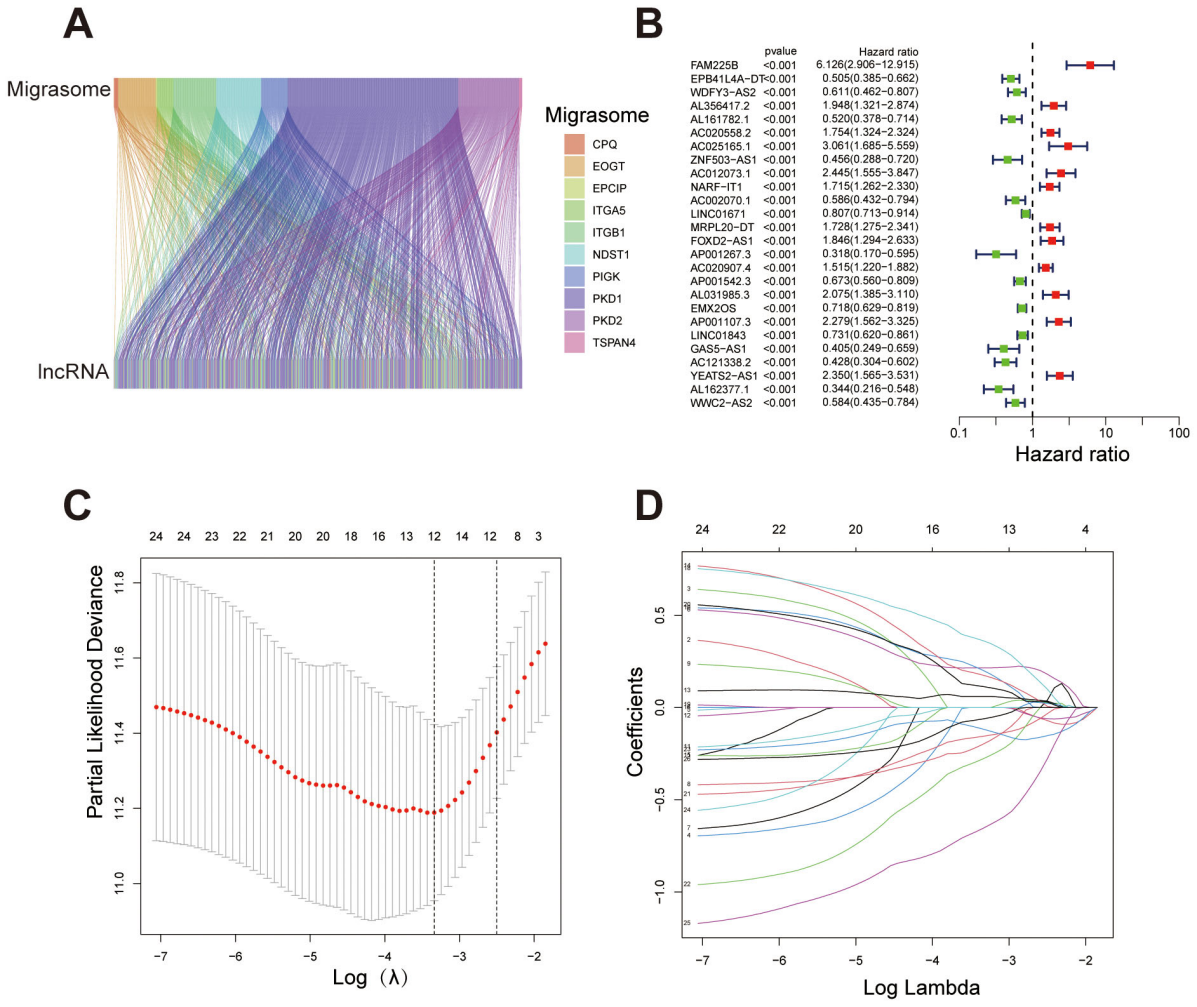
Identification of migrasome-related lncRNAs and construction of the prognostic signature. **(A)** Sankey diagram illustrating the association between migrasome-related genes and their co-expressed lncRNAs. **(B)** Forest plot showing lncRNAs significantly associated with overall survival in ccRCC patients (p < 0.001). Hazard ratios (HR) and 95% confidence intervals (CI) are displayed, with red indicating high-risk lncRNAs (HR > 1) and green indicating protective lncRNAs (HR < 1). **(C)** Lasso regression plot depicting the relationship between log(λ) and coefficients for selected variables, with numbers representing non-zero coefficients at different λ values. **(D)** Cross-validation plot determining the optimal λ value for Lasso regression. ***P < 0.001, **P < 0.01, *P < 0.05.

We identified migrasome-related genes through a literature review and by using GeneCards. When searching for relevant genes in GeneCards, we set the Relevance Score threshold to greater than 1. This process ultimately yielded eleven migrasome-related genes: ITGB1, ITGA5, EOGT, CPQ, PIGK, NDST1, TSPAN4, EPCIP, PKD2, PKD1, and TMX2-CTNND1 (4, 7, 14, 15). We obtained transcriptomic data for ccRCC from the TCGA database, generating an expression matrix from 542 tumor samples and 72 normal samples. The mRNA expression levels of migrasome-related genes were extracted and analyzed. We then identified co-expressed lncRNAs by filtering for those with an absolute correlation coefficient > 0.4 and a p-value < 0.001 in relation to migrasome-related genes (**Figure 1A**). The dataset was randomly divided into two groups: a training set and a testing set. We compared the data from the training and testing sets and confirmed that there were no statistically significant differences in clinical characteristics between the two groups (**Supplementary Table S2**). Univariate Cox regression analysis was performed in the training set to identify lncRNAs significantly associated with patient survival (**Figure 1B**). Next, a Lasso regression analysis was conducted to build the Lasso signature with cross-validation, and these selected lncRNAs were then used for multivariate Cox regression to construct the prognostic signature (**Figures 1C, D**). The signature was further refined using the stepwise selection method to obtain the final form. The resulting formula for the risk score is as follows: Risk score = -0.350867936624671 * ZNF503-AS1 + 0.635684241536826 * NARF-IT1 + 0.329706961392795 * FOXD2-AS1 + 0.54579941265602 * AL031985.3 - 0.28798278420868 * LINC01843 - 0.593407946785319 * GAS5-AS1 - 0.917182162566296 * AL162377.1.

**Figure 2:**
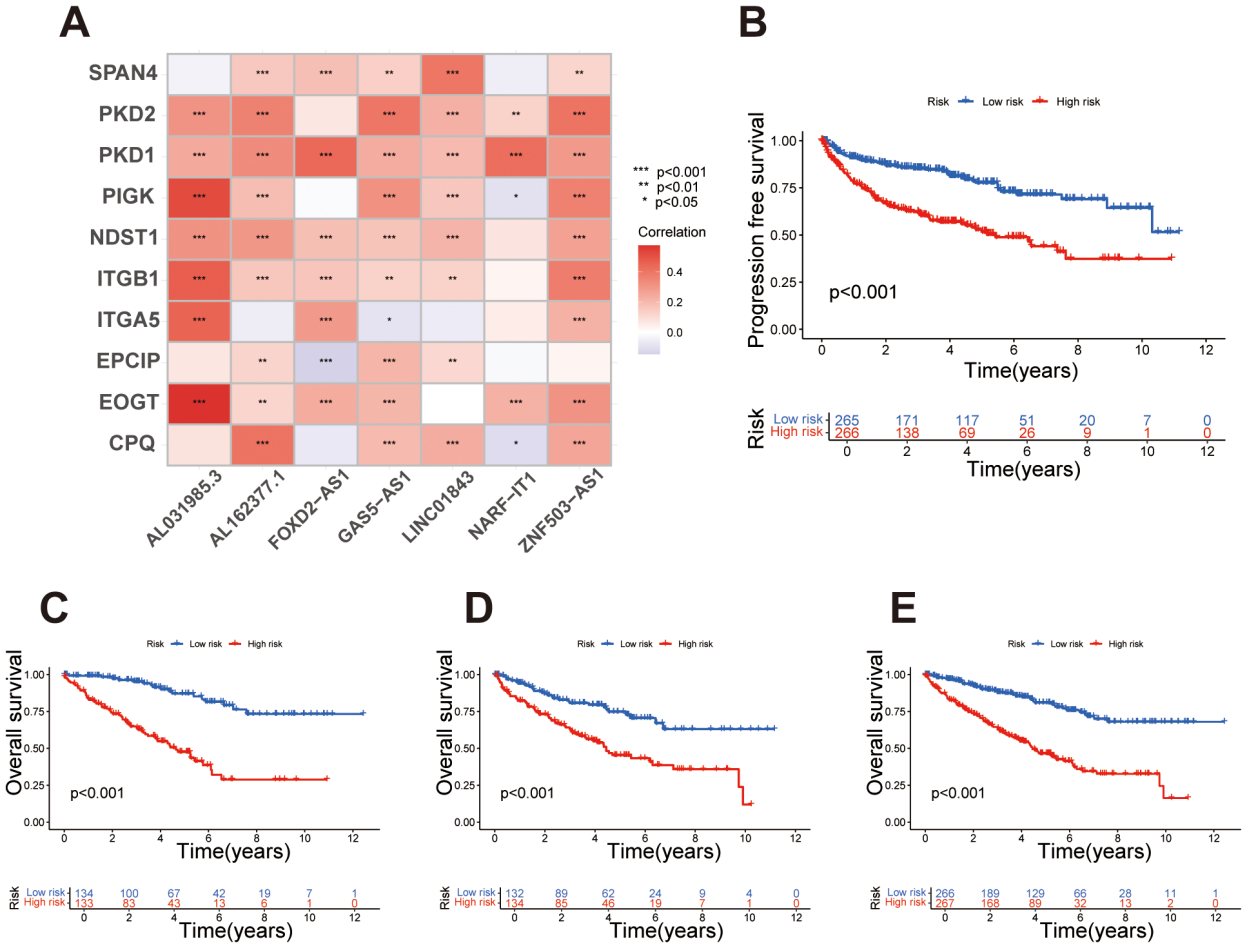
Survival analysis and correlation of the migrasome-related lncRNA signature. **(A)** Heatmap illustrating the correlation between migrasome-related genes and signature lncRNAs, with colors indicating Pearson correlation coefficients. **(B)** Kaplan-Meier survival curve for progression-free survival (PFS) in high-risk and low-risk groups, with statistical significance assessed using the log-rank test. **(C)** Kaplan-Meier survival curves for overall survival (OS) in the training set, comparing high-risk and low-risk groups based on the migrasome-related lncRNA signature. **(D)** Kaplan-Meier survival curves for OS in the testing set, stratifying samples into high-risk and low-risk groups. **(E)** Kaplan-Meier survival curves for OS in the all dataset, highlighting significant survival differences between risk groups.

After developing the prognostic signature formula in the training set, we validated the signature using the testing set. In the training set, risk scores for each sample were calculated according to the signature, and samples were classified into high- and low-risk groups based on the median risk score. In the testing set, risk scores were similarly calculated, and samples were grouped into high- and low-risk categories using the median risk score derived from the training set. As previously mentioned, chi-square test results showed that the p-values for variables such as gender, age, grade, and stage were all greater than 0.05 in both the training and testing sets, indicating no statistically significant differences between the two groups regarding these clinical characteristics. This result supports the homogeneity of the data across the sets. The balanced distribution of clinical characteristics between the two groups helps to minimize confounding effects from the outset. Next, we further explored the correlation between migrasome-related genes and signature lncRNAs. The results demonstrated varying levels of association, indicating an overall strong correlation between migrasome-related genes and the signature lncRNAs (**Figure 2A**). The Kaplan-Meier survival analysis based on the migrasome-related lncRNA signature revealed significant differences in overall survival (OS) and progression-free survival (PFS) between high-risk and low-risk groups. PFS analysis indicated that patients in the high-risk group had a significantly shorter PFS than those in the low-risk group, reinforcing the signature’s association with poor prognosis (**Figure 2B**). In the training set (**Figure 2C**), the high-risk group showed a notably shorter OS compared to the low-risk group (p < 0.001). This trend was consistently observed in the testing set and the combined all dataset, further validating the predictive power of the signature for OS (**Figures 2D, E**). These results underscore the strong prognostic value of the migrasome-related lncRNA signature.

**Figure 3:**
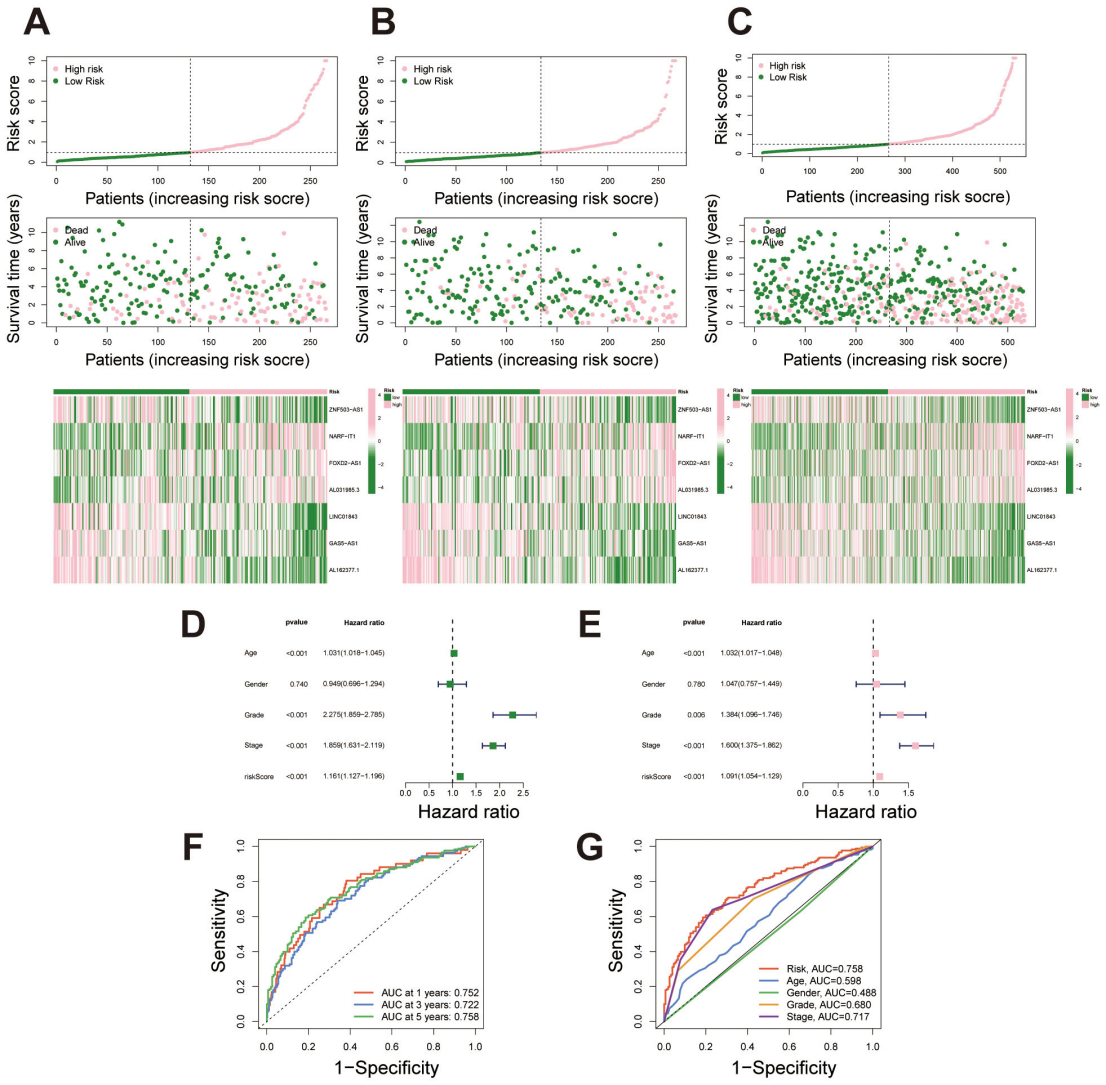
Risk stratification and predictive performance of the migrasome-related lncRNA signature. **(A-C)** Risk score distribution, survival status, and lncRNA expression heatmap for the training set, testing set, and combined dataset, with samples ordered by increasing risk score and stratified into high- and low-risk groups. **(D)** Univariate Cox regression analysis displaying hazard ratios (HR) and p-values for survival outcomes across clinical factors and the migrasome-related lncRNA signature. **(E)** Multivariate Cox regression analysis showing adjusted HR and p-values for significant clinical factors and the migrasome-related lncRNA signature, highlighting its independent prognostic value. **(F)** Time-dependent ROC curves at 1, 3, and 5 years for the migrasome-related lncRNA signature, demonstrating strong predictive accuracy for survival outcomes. **(G)** Comparative ROC curves for 5-year survival prediction, evaluating the signature against clinical factors including age, gender, grade, and stage.

Based on the prognostic signature, samples were ordered from left to right by increasing risk score and stratified into high- and low-risk groups using the median score as a threshold. Survival status plots revealed that the high-risk group had a markedly higher number of deceased samples compared to the low-risk group. Concurrently, expression heatmaps showed distinct differences in lncRNA expression levels between high- and low-risk groups, consistently observed across the training, test, and full datasets. These findings underscore the prognostic value of the lncRNA signature in stratifying patient risk (**Figures 3A-C**). Univariate Cox regression analysis showed that several clinical factors, including age, grade, stage, and the lncRNA-based risk score, were associated with survival outcomes in the dataset. Significant factors (p < 0.05) were then included in the multivariate Cox regression model to assess their independent prognostic value (**Figure 3D**). The multivariate analysis further confirmed that the lncRNA-based signature can serve as an independent prognostic factor, separate from other clinical characteristics such as age, grade, and stage, and is significantly associated with patient outcomes (**Figure 3E**). This further controls for the potential impact of confounding factors on the model outcomes. We conducted ROC analysis at 1, 3, and 5 years, finding that the migrasome-related lncRNA risk score demonstrated strong predictive accuracy for survival outcomes (**Figure 3F**). When compared with other clinical factors, including age, gender, grade, and stage, our prognostic signature outperformed these factors as an independent predictor of patient survival, highlighting its superior predictive power (**Figure 3G**).

**Figure 4:**
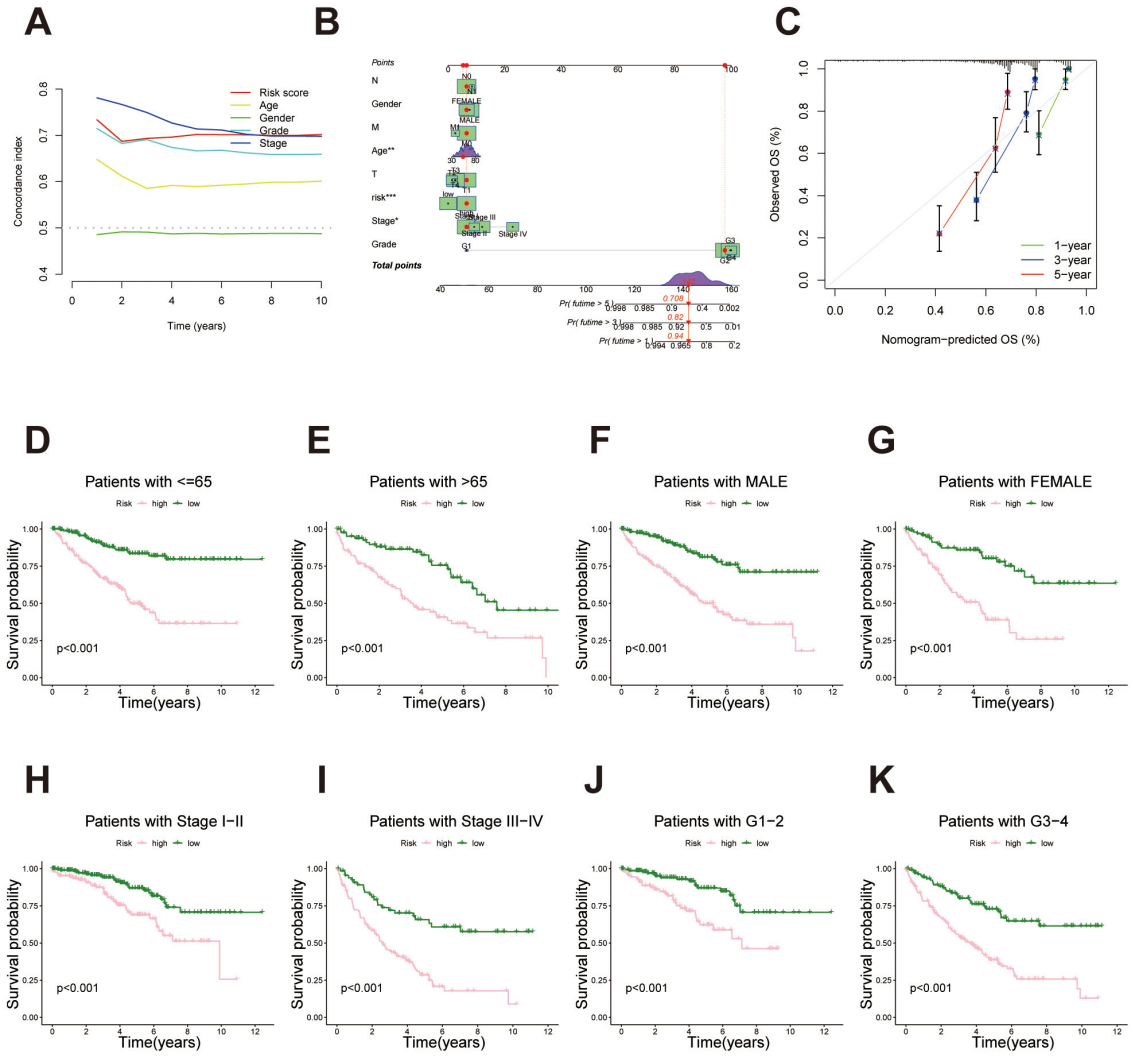
Survival analysis of the migrasome-related lncRNA signature across clinical subgroups. **(A)** C-index comparison between the lncRNA-derived prognostic signature and traditional clinicopathological features (age, gender, stage and tumor grade), indicating enhanced predictive power of the proposed model. **(B)** Construction of a prognostic nomogram combining the risk score and clinical factors for individualized prediction of 1-, 3-, and 5-year overall survival. **(C)** Calibration curves evaluating the concordance between predicted survival probabilities and actual outcomes, underscoring the nomogram’s reliability. **(D, E)** Kaplan–Meier survival curves for age subgroups (≤65 years and >65 years) demonstrate significantly poorer survival outcomes for high-risk patients in both categories. **(F, G)** Kaplan–Meier survival curves for gender subgroups (male and female) demonstrate significantly poorer survival outcomes for high-risk patients in both categories. **(H, I)** Kaplan–Meier survival curves for stage-based subgroups (stages I-II and stages III-IV) demonstrate significantly poorer survival outcomes for high-risk patients in both categories. **(J, K)** Kaplan–Meier survival curves for tumor grade-based subgroups (G1–2 and G3-4) demonstrate significantly poorer survival outcomes for high-risk patients in both categories.

To assess the predictive performance of the migrasome-related lncRNA signature, we calculated the concordance index (C-index) for the risk score and conventional clinical factors. The risk score yielded a favorable C-index value, indicating strong prognostic capability in overall survival prediction (**Figure 4A**). A nomogram combining the risk score with clinical variables was developed to enable individualized survival prediction (**Figure 4B**). Calibration plots showed strong concordance between predicted and actual 1-, 3-, and 5-year survival outcomes, confirming the nomogram’s reliability (**Figure 4C**). We further investigated the predictive power of the migrasome-related lncRNA risk signature across different clinical subgroups. First, in patients stratified by age (≤65 years and >65 years), the signature classified each group into high- and low-risk categories. Results showed that in both age groups, the high-risk patients had significantly poorer survival outcomes than the low-risk group (**Figures 4D, E**). Similarly, in the gender subgroups, both male and female patients in the high-risk category exhibited significantly worse survival than those in the low-risk category (**Figures 4F, G**). In stages I-II and III-IV, survival rates were consistently lower in the high-risk group across both stages, further validating the prognostic power of the signature across different clinical stages (**Figures 4H, I**). Finally, for tumor grade, patients were grouped into G1–2 and G3–4 categories. The high-risk group showed markedly poorer survival outcomes compared to the low-risk group (**Figures 4J, K**).

**Figure 5:**
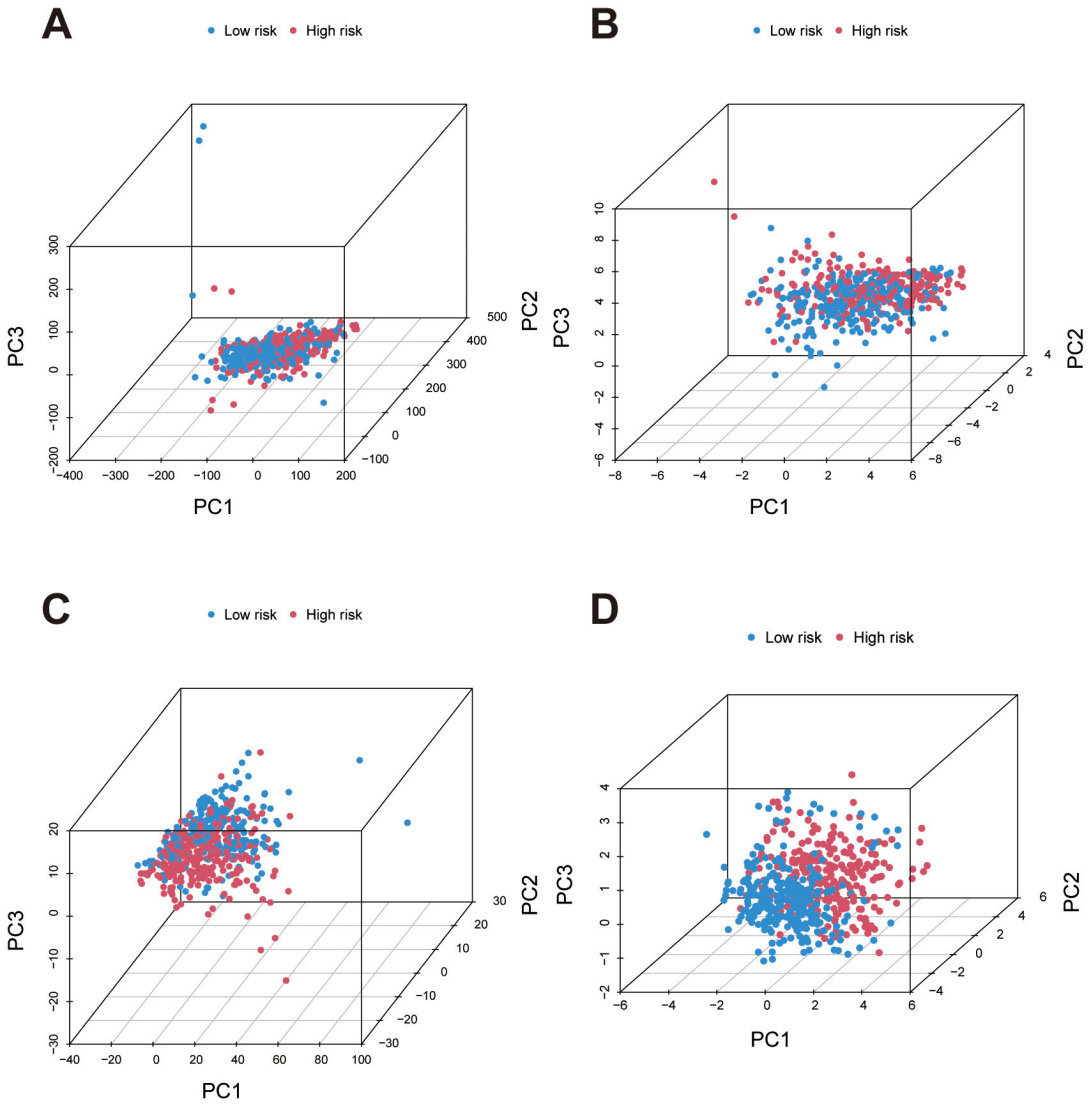
Principal Component Analysis (PCA) for risk group separation based on different gene sets. **(A)** PCA plot using all genes shows minimal separation between high- and low-risk groups, indicating low discriminatory power. **(B)** PCA plots based on migrasome-related genes demonstrate improved clustering of high- and low-risk groups, highlighting their stronger association with risk stratification. **(C)** PCA plots based on migrasome-related lncRNAs demonstrate improved clustering of high- and low-risk groups, highlighting stronger association with risk stratification. **(D)** PCA using lncRNAs from the prognostic signature achieves the most distinct separation between high- and low-risk groups, confirming the effectiveness of the signature in risk differentiation.

Principal Component Analysis (PCA) was performed on four data sets to assess the distribution of high- and low-risk patients based on different gene sets. First, in the analysis using all genes, there was no clear separation between high- and low-risk groups, indicating that the overall gene expression profile was insufficient to effectively distinguish these risk categories (**Figure 5A**). In contrast, PCA based on migrasome-related genes and migrasome-related lncRNAs showed relatively clearer clustering of high- and low-risk groups, suggesting that these genes have a stronger association with risk stratification (**Figures 5B, C**). Finally, PCA using the risk lncRNAs from our prognostic signature revealed a distinct separation between high- and low-risk groups, further validating the effectiveness of this signature for risk stratification (**Figure 5D**).

### 3.2 Functional enrichment analysis highlights divergent biological pathways between risk subgroups in ccRCC

**Figure 6:**
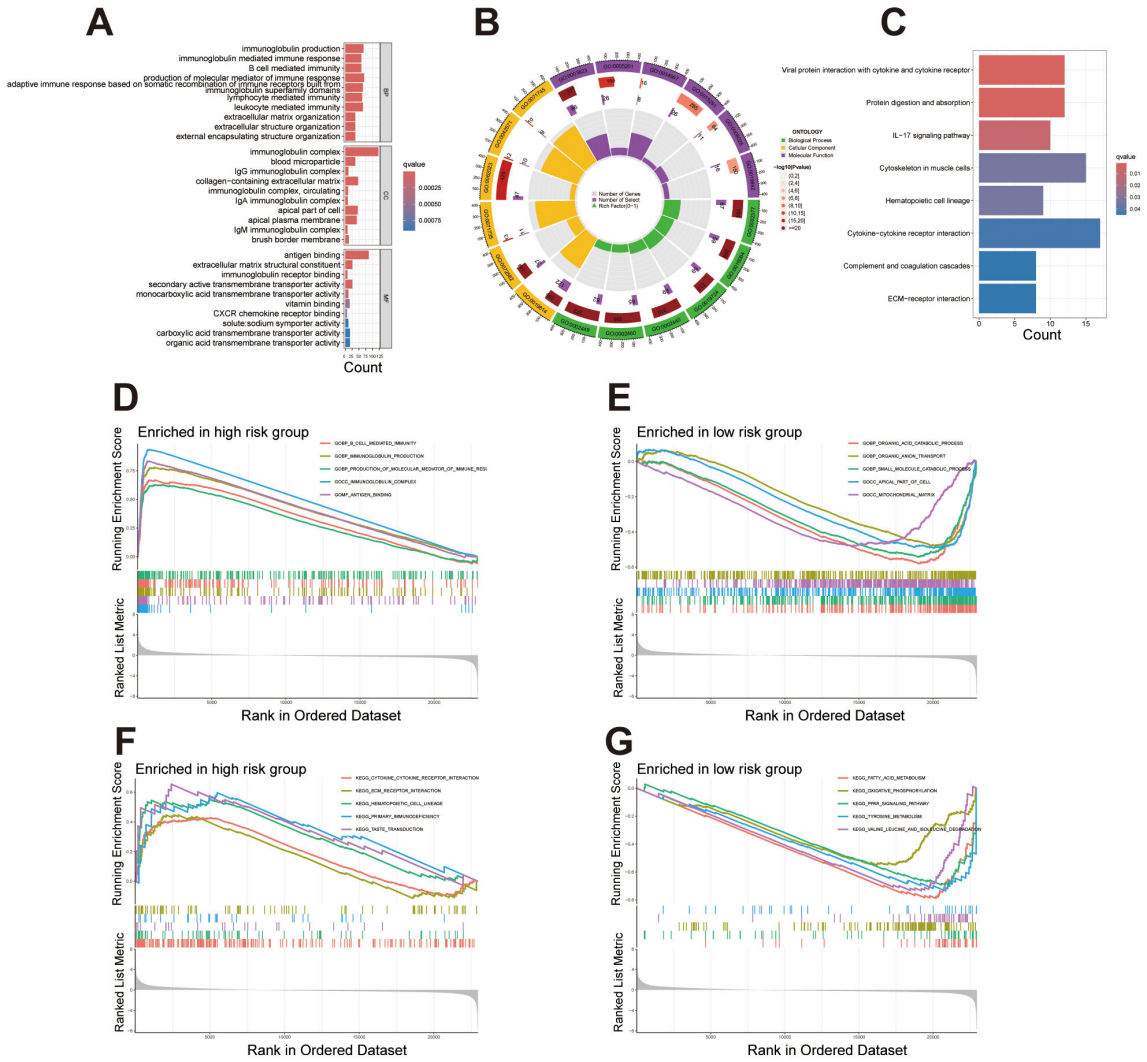
Enrichment analysis reveals functional and pathway differences between high- and low-risk groups. **(A)** Bar plot showcasing the top 10 enriched terms across GO categories (BP, CC, MF), emphasizing immune response, extracellular matrix organization, and transmembrane transport. **(B)** Circular plot illustrating gene proportions and enrichment significance for selected GO terms in each category. **(C)** KEGG pathway enrichment analysis displaying differentially expressed genes involved in key biological pathways. **(D, E)** GSEA plots presenting the top five enriched functions in the high-risk and low-risk groups. **(F, G)** GSEA plots presenting the top five enriched pathways in the high-risk and low-risk groups.

GO enrichment analysis revealed significant enrichment of differentially expressed genes within biological processes (BP), cellular components (CC), and molecular functions (MF), primarily involving immune response, extracellular matrix organization, and transmembrane transport (**Figure 6A**). A circular plot further visualizes gene proportions across categories and KEGG pathway enrichment analysis identified significant enrichment in pathways including cytokine-cytokine receptor interaction, IL-17 signaling, complement and coagulation cascades, and extracellular matrix (ECM) receptor interaction (**Figures 6B, C**). These pathways suggest an association of the identified genes with immune response regulation, cell signaling, and tissue structural integrity. We conducted GSEA analysis to identify significant functional enrichment in the high and low-risk groups. The results revealed that the high-risk group exhibited enrichment in functions related to immune responses, such as B-cell mediated immunity, immunoglobulin production, and antigen binding (**Figure 6D**). In contrast, the low-risk group showed enrichment in functions associated with metabolic processes and cellular structures, including organic acid catabolism and mitochondrial matrix localization (**Figure 6E**). These findings indicate that the migrasome-related lncRNA signature is linked to distinct biological functions across risk categories. The pathway-based GSEA analysis revealed significant enrichment in the high-risk group for pathways related to cytokine-cytokine receptor interaction, extracellular matrix-receptor interaction, hematopoietic cell lineage, primary immunodeficiency, and taste transduction, indicating a strong association with immune processes (**Figure 6F**). Conversely, pathways enriched in the low-risk group primarily involved fatty acid metabolism, oxidative phosphorylation, PPAR signaling, tyrosine metabolism, and branched-chain amino acid metabolism, highlighting metabolic functional differences (**Figure 6G**).

### 3.3 Comprehensive characterization of the immune microenvironment and tumor mutation burden in ccRCC risk groups

**Figure 7:**
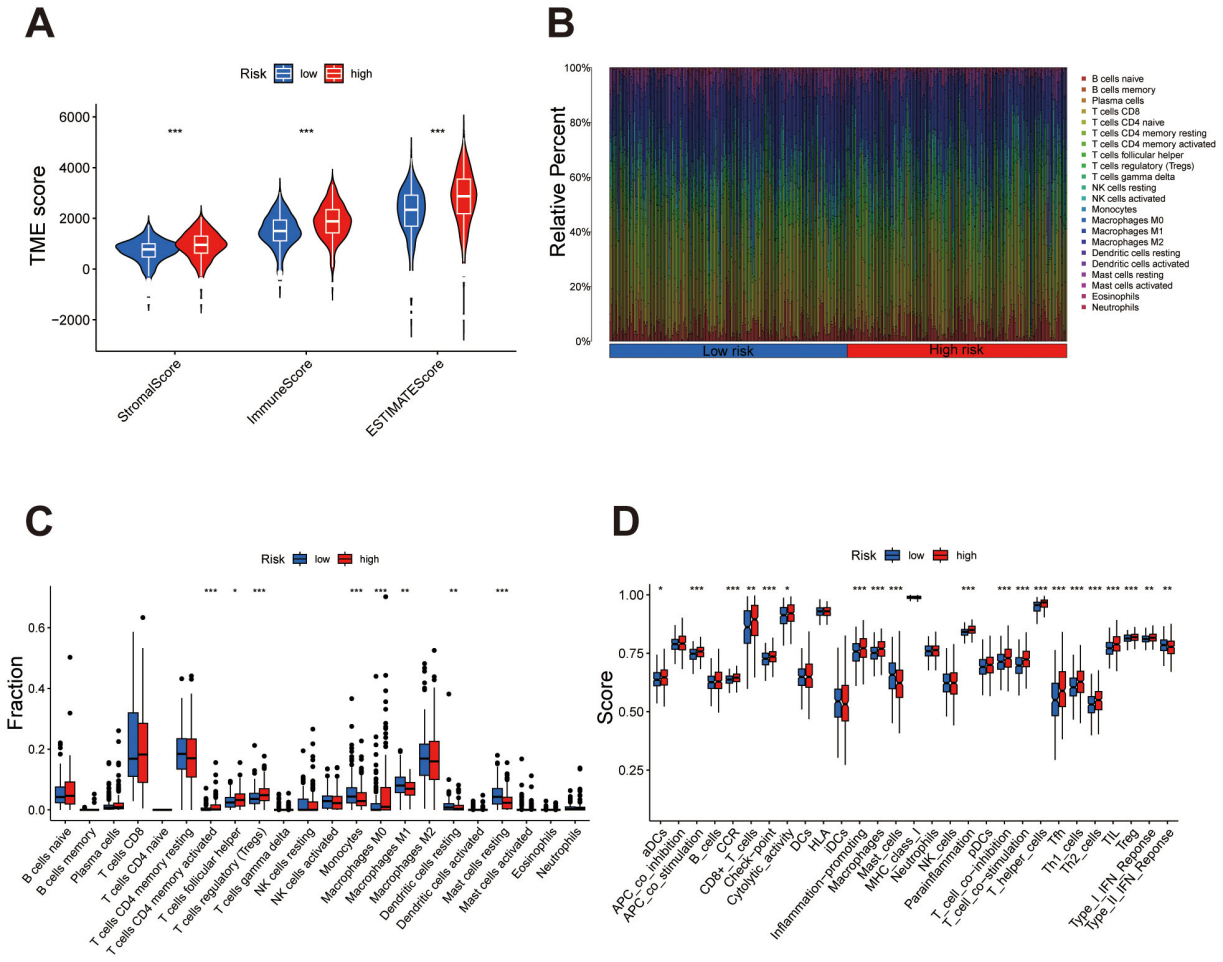
Immune microenvironment characteristics differ between high- and low-risk groups. **(A)** Violin plot comparing StromalScore, ImmuneScore, and ESTIMATEScore, with higher scores consistently observed in the high-risk group. **(B)** Bar plot showing the relative abundance of immune cell types, highlighting differences in immune composition between the groups. **(C)** Box plot quantifying significant differences in immune cell subsets, emphasizing specific cell types enriched in high- or low-risk groups. **(D)** Box plot demonstrating notable differences in immune functions, underscoring elevated immune activity or suppression in high-risk versus low-risk groups. ***P < 0.001, **P < 0.01, *P < 0.05.

The violin plot analysis compared TME scores between high- and low-risk groups, covering the StromalScore, ImmuneScore, and overall ESTIMATEScore. Results indicated that each TME score was elevated in the high-risk group relative to the low-risk group, suggesting a greater presence of stromal and immune cells within the high-risk group (**Figure 7A**). Immune cell subset distributions between high- and low-risk groups are compared. The bar plot shows that the high-risk group exhibits a higher relative abundance of immune cells such as regulatory T cells (Tregs) and M0 macrophages, whereas the low-risk group displays an increased abundance of immune cells like M1 macrophages and resting mast cells (**Figure 7B**). The box plot further validates these patterns, highlighting significant differences in immune cell subsets between the risk groups (**Figure 7C**). The immune function analysis identified notable differences between high- and low-risk groups, particularly in specific immune responses and cell activity. Functions such as APC co-stimulation, cytolytic activity, and inflammation promotion were significantly elevated in the high-risk group. These findings underscore distinct immune activation and regulatory mechanisms between the two groups (**Figure 7D**).

**Figure 8:**
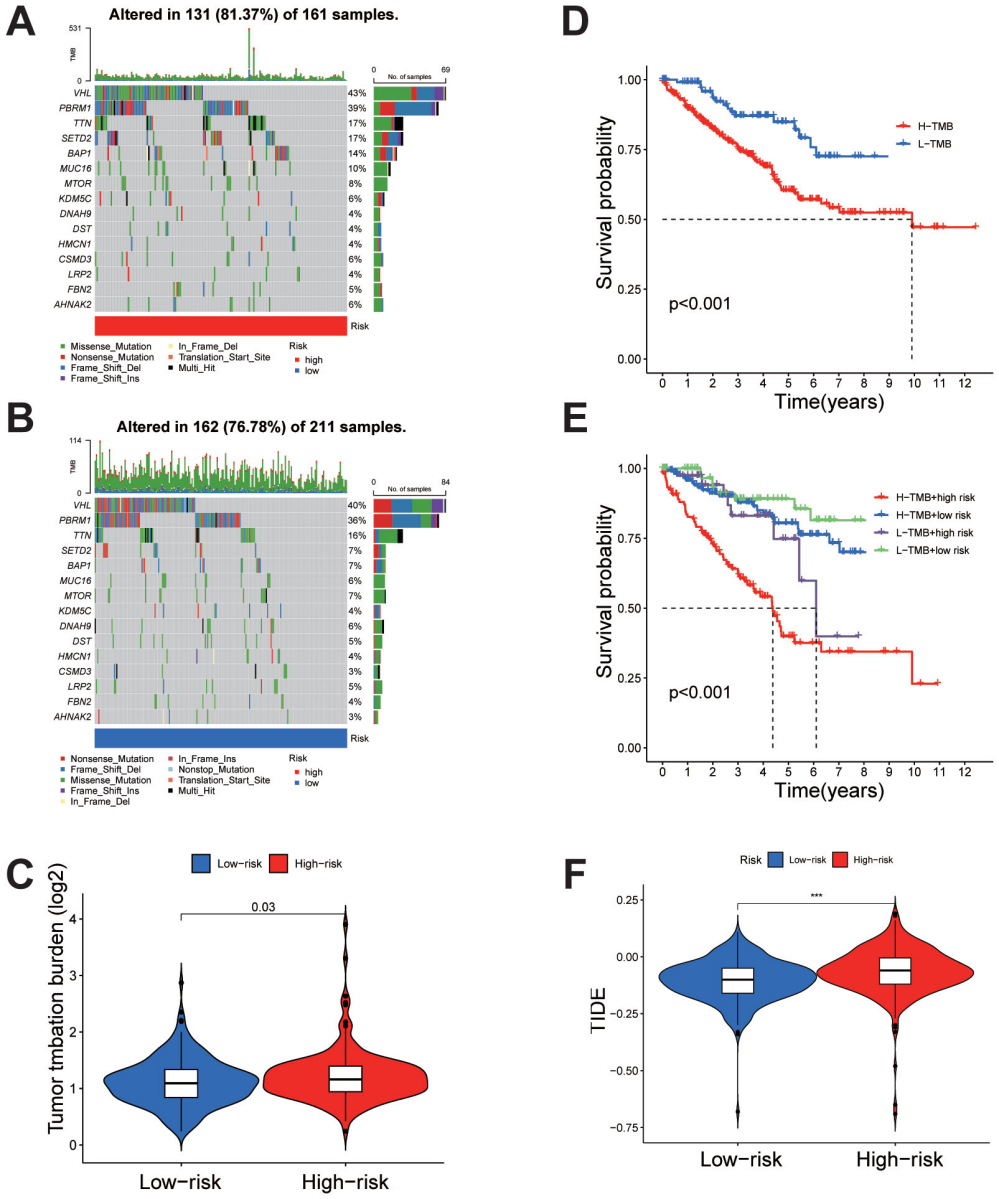
Tumor mutation burden (TMB) and immune evasion differences between high- and low-risk groups. **(A, B)** Comparison of TMB in high-risk and low-risk groups, respectively, showing higher mutation rates in the high-risk group. **(C)** Overall analysis indicating significantly elevated TMB in the high-risk group compared to the low-risk group. **(D)** Kaplan-Meier survival analysis comparing high TMB (H-TMB) and low TMB (L-TMB) groups, with poorer survival observed in the H-TMB group. **(E)** Combined analysis of TMB status and risk stratification reveals the lowest survival rates in the H-TMB + high-risk subgroup and the highest in the L-TMB + low-risk subgroup. **(F)** TIDE score comparison between high-risk and low-risk groups, demonstrating significantly higher immune evasion potential in the high-risk group. ***P < 0.001.

The mutation profiles of the top 15 most frequently mutated genes were analyzed separately for high- and low-risk groups. In the high-risk group, genes such as VHL and PBRM1 show high mutation frequencies (**Figure 8A**), while in the low-risk group, significant mutations in these genes are also observed (**Figure 8B**). Overall, the mutation burden is notably higher in the high-risk group compared to the low-risk group (**Figures 8A-C**). In the high TMB (H-TMB) group, overall survival was lower than in the low TMB (L-TMB) group (**Figure 8D**). Combining TMB status with risk stratification revealed that patients in the H-TMB + high-risk subgroup had the lowest survival rates, while those in the L-TMB + low-risk subgroup showed the highest survival, highlighting the combined impact of these factors (**Figure 8E**). TIDE analysis revealed a significantly higher TIDE score in the high-risk group compared to the low-risk group (**Figure 8F**), suggesting a potentially enhanced immune evasion capacity within the high-risk group.

### 3.4 Differential drug sensitivity profiles between high- and low-risk groups in ccRCC

**Figure 9:**
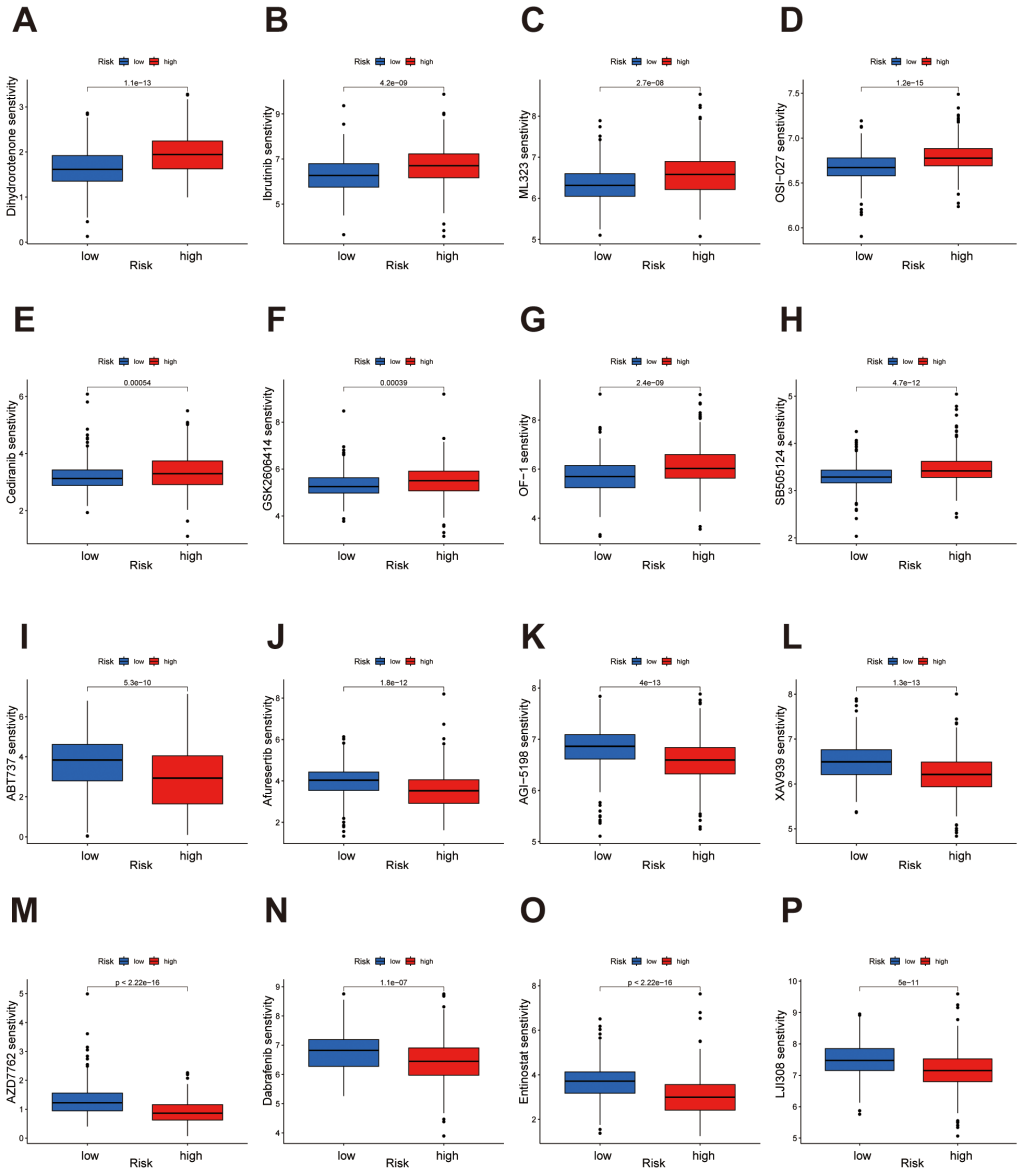
Drug sensitivity analysis highlights distinct responses in high- and low-risk groups. **(A-H)** The low-risk group demonstrates heightened sensitivity to Dihydrorotenone, Ibrutinib, ML323, OSI-027, Cediranib, GSK2606414, OF-1, and SB505124. **(I-P)** The high-risk group demonstrates heightened sensitivity to ABT737, Afuresertib, AGI-5198, XAV939, AZD7762, Dabrafenib, Entinostat, and LJI308.

Drug sensitivity analysis revealed significant differences in response to multiple drugs between high- and low-risk groups. In the low-risk group, drugs such as Dihydrorotenone, Ibrutinib, ML323, OSI-027, Cediranib, GSK2606414, OF-1 and SB505124 demonstrated higher sensitivity (**Figures 9A-H**). Conversely, in the high-risk group, drugs including ABT737, Afuresertib, AGI-5198, XAV939, AZD7762, Dabrafenib, Entinostat and LJI308 showed enhanced sensitivity (**Figures 9I-P**). Comprehensive results are presented in the **Supplementary Figures S1** and **S2**. These findings provide potential guidance for individualized therapeutic strategies across risk groups, indicating that specific drugs may yield distinct efficacy depending on the risk category.

### 3.5 Single-cell transcriptomic analysis reveals cellular heterogeneity and signature-associated tumor microenvironmental features in ccRCC

**Figure 10:**
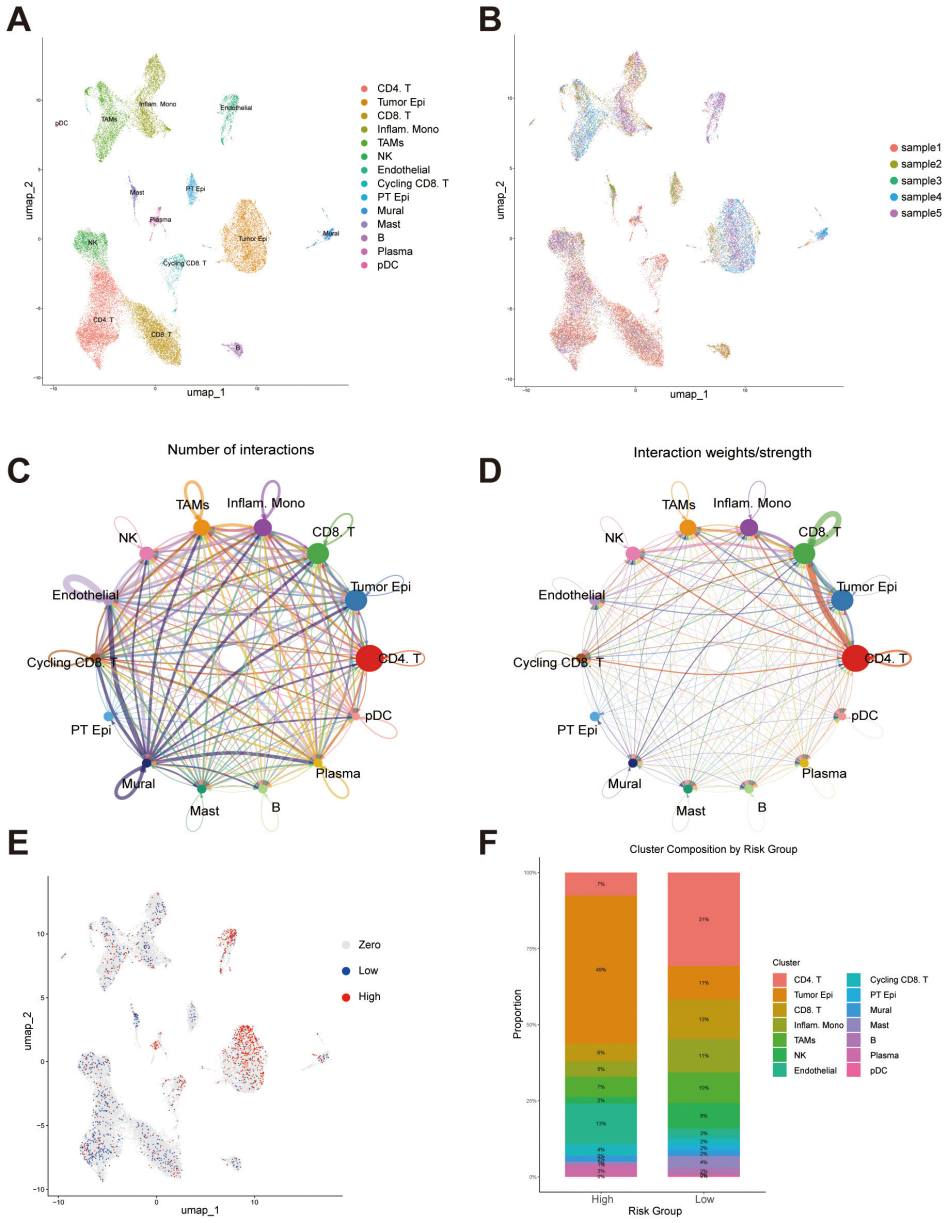
Single-cell transcriptomic analysis reveals cellular composition, intercellular communication, and risk score distribution. **(A)** UMAP plot showing the distribution of major cell populations identified across all samples, including CD4^+^ T cells, CD8^+^ T cells, tumor epithelial cells, inflammatory monocytes, tumor-associated macrophages (TAMs), NK cells, endothelial cells, cycling CD8^+^ T cells, proximal tubular epithelial (PT Epi) cells, mural cells, mast cells, B cells, plasma cells, and plasmacytoid dendritic cells (pDCs). **(B)** UMAP visualization colored by individual sample. **(C)** Intercellular communication network illustrating the number of predicted interactions among different cell types. **(D)** Intercellular communication network weighted by interaction strength. **(E)** UMAP plot displaying the distribution of signature scores at the single-cell level. **(F)** Bar plot showing the proportion of each cell type within the high- and low-risk groups.

To further explore the capacity of our constructed prognostic signature to distinguish cellular heterogeneity and intercellular communication patterns within the tumor microenvironment of ccRCC, we analyzed publicly available single-cell RNA sequencing (scRNA-seq) data. Following standard quality control, normalization, and dimensionality reduction procedures, UMAP visualization revealed distinct clustering of major cell populations, including CD4^+^ T cells, tumor epithelial cells, CD8^+^ T cells, inflammatory monocytes, tumor-associated macrophages (TAMs), NK cells, endothelial cells, cycling CD8^+^ T cells, proximal tubular epithelial cells, mural cells, mast cells, B cells, plasma cells, and plasmacytoid dendritic cells (pDCs) (**Figure 10A**). Cells from different patient samples were evenly distributed across clusters, indicating minimal batch effects after correction (**Figure 10B**). Cell-cell communication analysis demonstrated that CD4^+^ T cells, CD8^+^ T cells and tumor epithelial cells were central hubs in the interaction network, exhibiting the highest number of intercellular connections (**Figure 10C**). These two cell types also showed the greatest interaction strengths, suggesting they play critical roles in orchestrating the tumor microenvironment (**Figure 10D**). To investigate the relationship between the risk signature and the tumor ecosystem at single-cell resolution, we scored each cell based on the signature. To minimize the potential bias from low sequencing depth, we excluded cells with a score of zero. The remaining cells were then divided into high- and low-risk groups using the median score as a threshold. The distribution of signature expression across cell types revealed that high-risk cells were predominantly enriched in the tumor epithelial population (**Figure 10E**). Furthermore, the cellular composition differed markedly between the high- and low-risk groups. The high-risk group displayed a greater proportion of tumor epithelial cells and endothelial cells, while CD4^+^ T cells were more abundant in the low-risk group (**Figure 10F**). These findings highlight distinct immunological and stromal characteristics between the two risk groups, implying that differences in the tumor microenvironment may underlie the divergent clinical outcomes associated with the risk signature.

**Figure 11:**
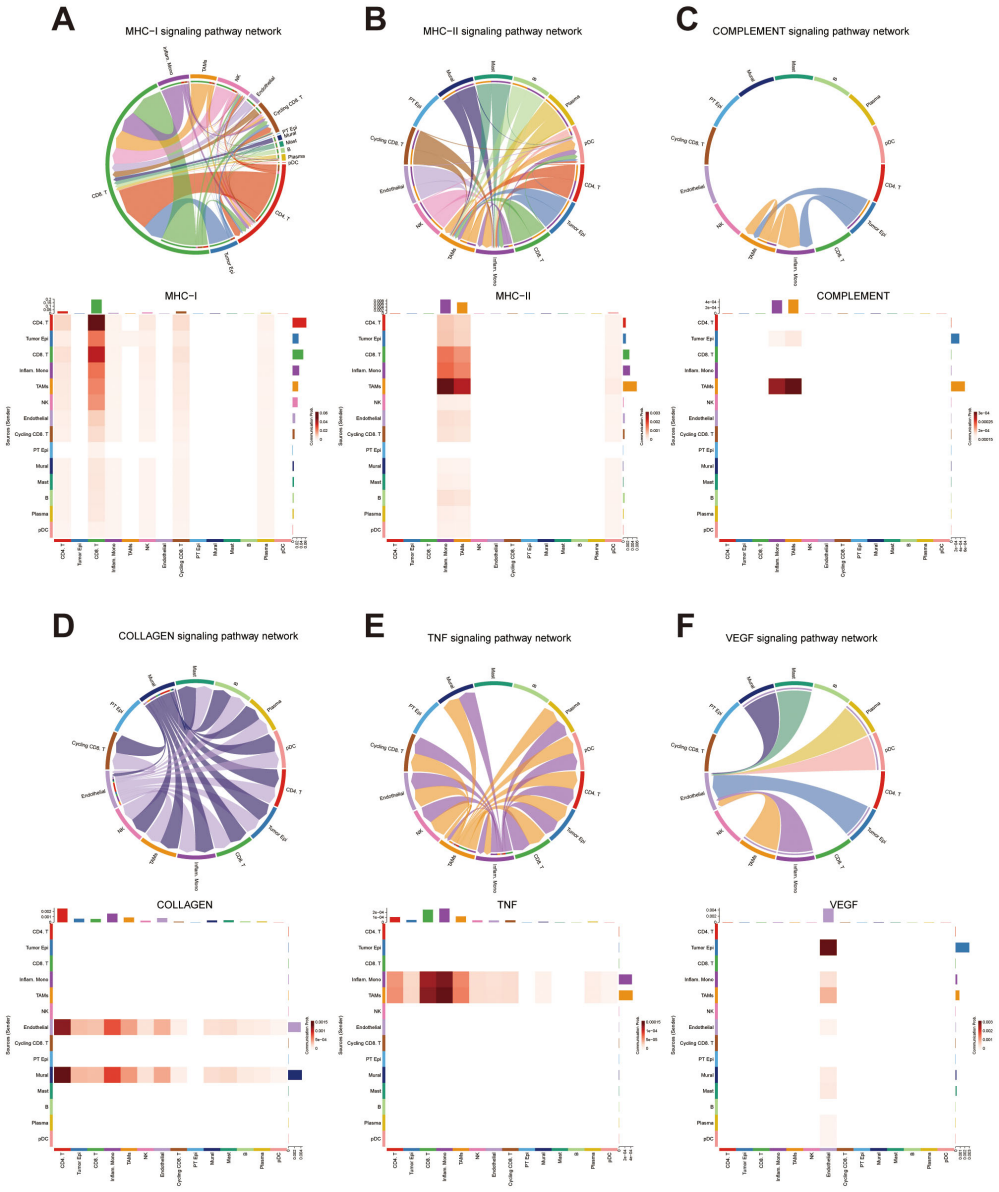
Intercellular Communication Analysis Based on Functional Enrichment Results **(A)** Intercellular communication network within the MHC class I signaling pathway. **(B)** Intercellular communication network within the MHC class II signaling pathway. **(C)** Intercellular communication network within the COMPLEMENT signaling pathway. **(D)** Intercellular communication network within the COLLAGEN signaling pathway. **(E)** Intercellular communication network within the TNF signaling pathway. **(F)** Intercellular communication network within the VEGF signaling pathway.

Building upon our previous functional enrichment results (**Figure 6**), we further explored how specific signaling pathways enriched are mediated through intercellular communication at the single-cell level. Given the enrichment of immune-related terms such as “GOBP_IMMUNE_SYSTEM_PROCESS” in the high-risk group, we focused on several key immune signaling pathways, including MHC I, MHC II, and the complement. These analyses revealed that tumor epithelial cells, CD8^+^ T cells, inflammatory monocytes, and TAMs serve as primary contributors to immune-related signaling, forming dense and directional interaction networks among these cell types (**Figures 11A-C**).

Additionally, the enrichment of “ECM_RECEPTOR_INTERACTION” prompted investigation of the collagen signaling pathway. We found that endothelial cells and mural cells were heavily involved in collagen-mediated signaling, primarily interacting with CD4^+^ T cells and inflammatory monocytes. These findings suggest a potential role of stromal–immune cell communication in shaping the extracellular matrix environment within the tumor (**Figure 11D**).

We also examined cytokine-related pathways based on the enrichment of “CYTOKINE_CYTOKINE_RECEPTOR_INTERACTION” in the high-risk group. In the TNF signaling network, inflammatory monocytes and TAMs emerged as major signal sources, communicating predominantly with CD8^+^ T cells and other monocytes. In contrast, VEGF signaling was characterized by frequent interactions between tumor epithelial cells and endothelial cells, indicating a strong tumor–vasculature crosstalk potentially involved in angiogenesis. Collectively, these results provide additional insight into the pathway-specific cellular interactions that may underlie the differential biological behavior of high-risk tumors (**Figures 11E, F**).

**Figure 12:**
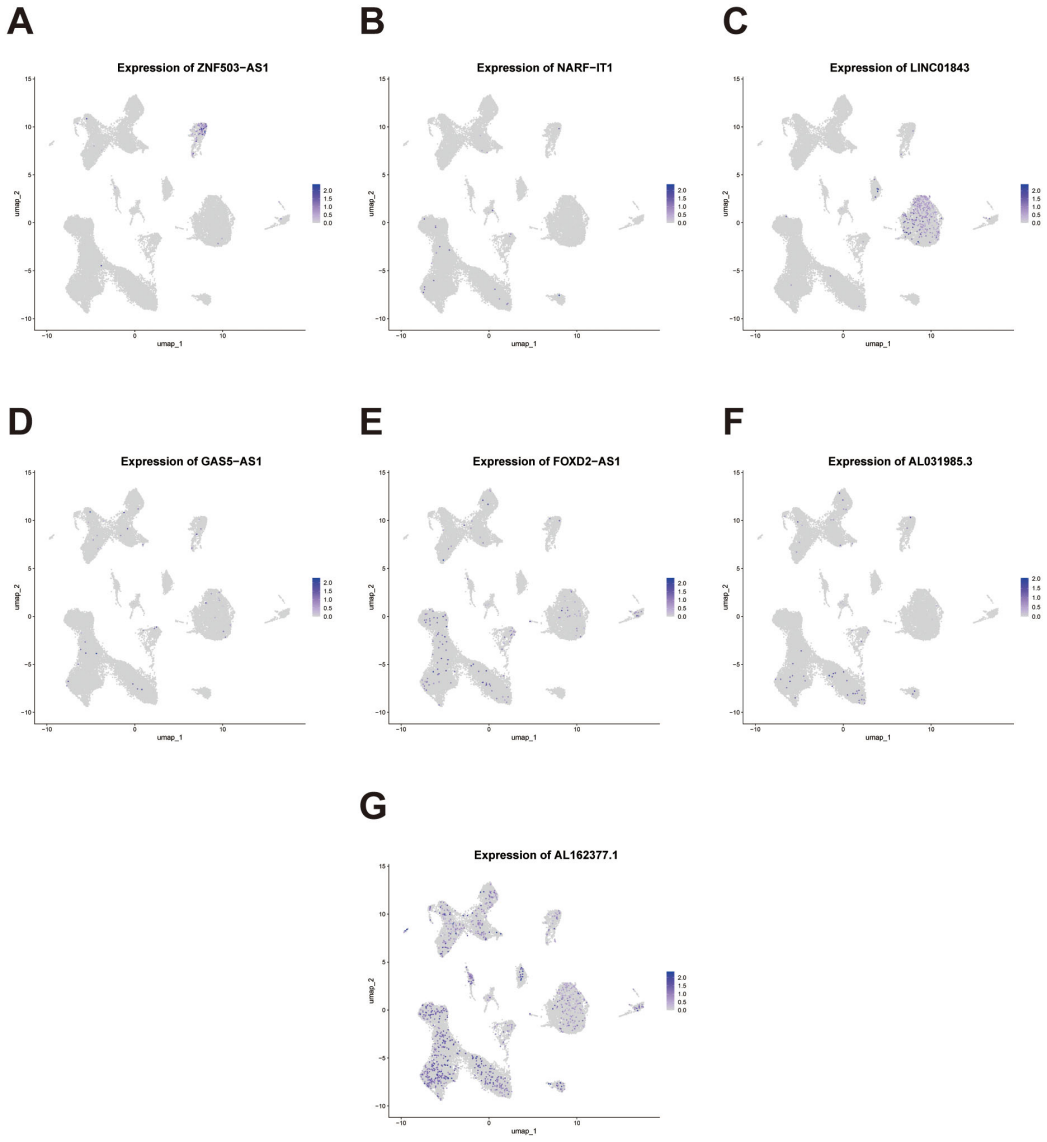
Single-cell expression patterns of the signature genes across cellular clusters. **(A-G)** Feature plots depicting the single-cell expression distribution of the seven signature lncRNAs (ZNF503-AS1, NARF-IT1, LINC01843, GAS5-AS1, FOXD2-AS1, AL031985.3, and AL162377.1) across distinct cell clusters.

To further investigate the cell-type–specific expression patterns of the seven genes comprising our prognostic signature, we performed FeaturePlot visualization using the integrated single-cell dataset. The results revealed heterogeneous expression across distinct cellular clusters (**Figures 12A-G**). Notably, ZNF503-AS1 exhibited enriched expression in endothelial cells, suggesting its potential involvement in aberrant angiogenesis or vascular-associated immune and inflammatory processes. LINC01843 was predominantly expressed in tumor epithelial cells, indicating a possible role in tumor-intrinsic biological functions. AL031985.3 showed preferential expression in T cell populations, implying its potential function in modulating the tumor immune microenvironment. In contrast, AL162377.1 displayed no strong cell-type specificity and was expressed across multiple clusters.

These divergent expression patterns, observed at single-cell resolution, underscore the biological relevance of the signature genes and support their capacity to reflect both tumor-intrinsic and immune microenvironmental characteristics in ccRCC.

### 3.6 Experimental validation of factors supporting their role in the prognostic signature

**Figure 13:**
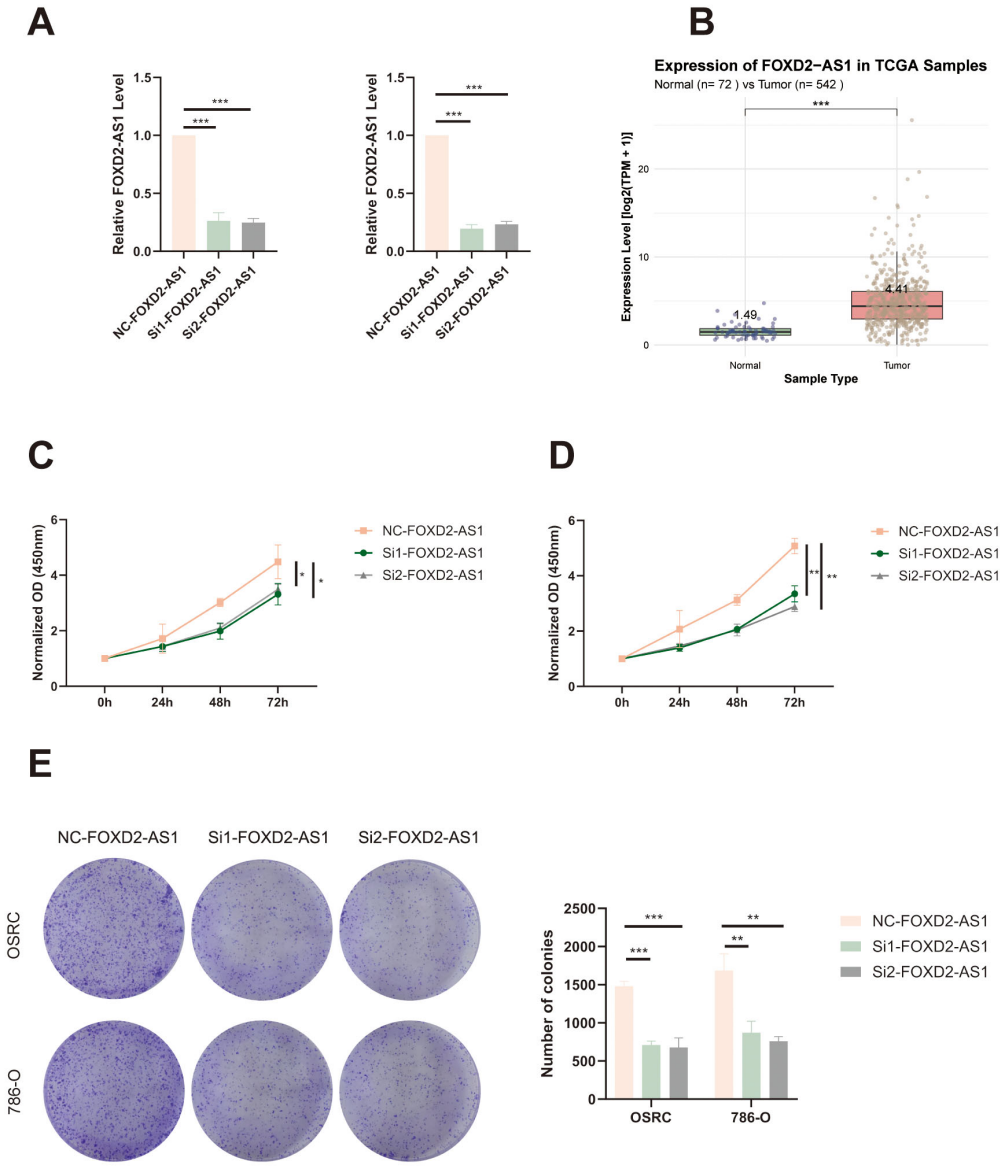
FOXD2-AS1 promotes proliferation of ccRCC cells. **(A)** qRT-PCR analysis showing the knockdown efficiency of FOXD2-AS1 in OS-RC-2 and 786-O cells after siRNA transfection. **(B)** The expression level of FOXD2-AS1 in ccRCC tissues and adjacent normal tissues based on TCGA data. **(C, D)** CCK-8 assays demonstrating that silencing FOXD2-AS1 significantly suppresses the proliferation of OS-RC-2 and 786-O cells over time. **(E)** Colony formation assay showing a marked reduction in the number and size of colonies formed by FOXD2-AS1-silenced cells compared to controls. ***P < 0.001, **P < 0.01, *P < 0.05.

To further validate and reinforce the reliability of our migrasome-associated lncRNA prognostic signature, we performed a series of functional experiments focusing on FOXD2-AS1, one of the key components of the model. The knockdown efficiency of FOXD2-AS1 was confirmed by qRT-PCR in OS-RC-2 and 786-O cell lines, showing a significant reduction in expression levels following siRNA transfection compared with the negative control group (**Figure 13A**). In addition, analysis of TCGA data revealed that FOXD2-AS1 was significantly upregulated in ccRCC tissues compared to adjacent normal tissues. (**Figure 13B**). We next investigated whether FOXD2-AS1 influences cell proliferation. CCK-8 assays revealed that FOXD2-AS1 silencing led to a marked decrease in the proliferative capacity of tumor cells (**Figures 13C, D**). Consistently, colony formation assays showed that knockdown of FOXD2-AS1 significantly impaired the ability of cells to form colonies (**Figure 13E**).

**Figure 14:**
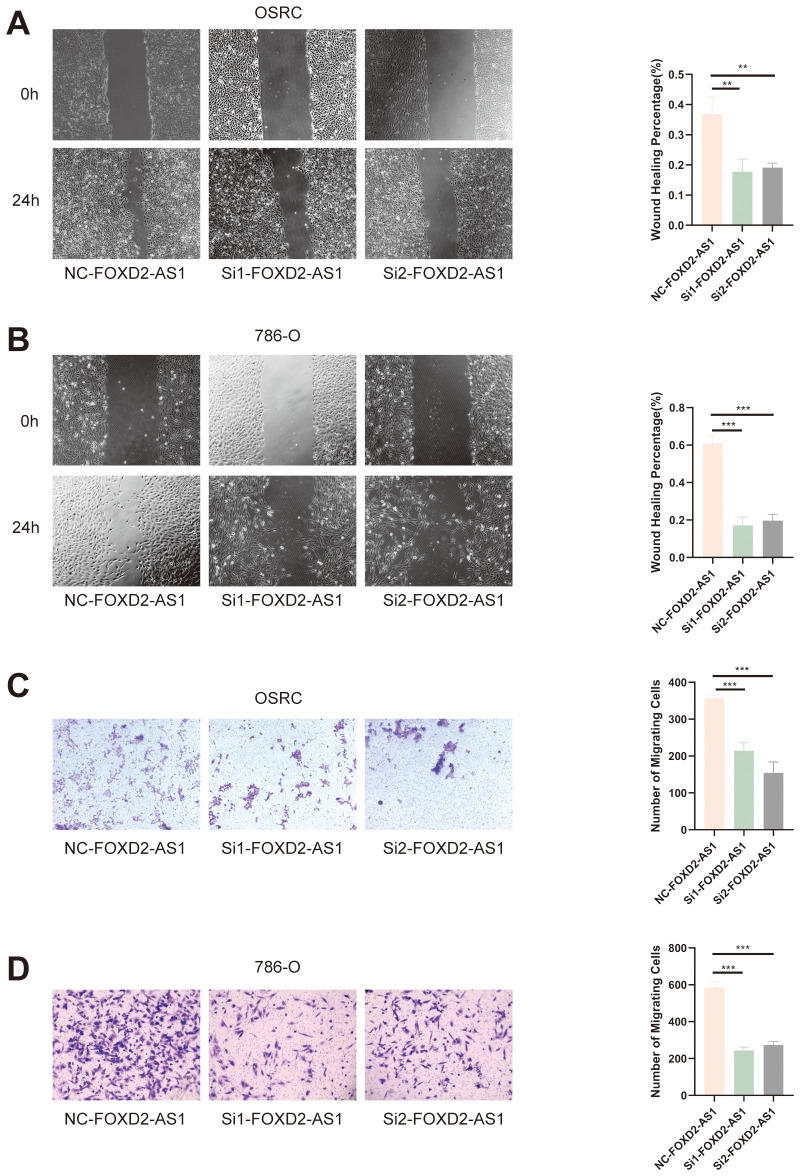
FOXD2-AS1 facilitates ccRCC cell migration. **(A, B)** Wound healing assay showing that FOXD2-AS1 knockdown impairs cell migration, as indicated by reduced wound closure after 24 hours. **(C, D)** Transwell migration assay further confirming that FOXD2-AS1 silencing significantly decreases the number of migrating OS-RC-2 and 786-O cells. ***P < 0.001, **P < 0.01.

To assess the effect of FOXD2-AS1 on cell migration, we performed wound healing and Transwell assays. The wound healing assay demonstrated that FOXD2-AS1 knockdown markedly suppressed cell migratory activity, as indicated by reduced wound closure after 24 hours (**Figures 14A, B**). Similarly, the Transwell migration assay showed a substantial decrease in the number of migrating cells in the FOXD2-AS1 knockdown group compared to the control (**Figures 14C, D**). Meanwhile, we also evaluated the proliferative and migratory effects of two other key factors in the signature, ZNF503-AS1 and GAS5-AS1, using the same experimental approach. The results demonstrated that both lncRNAs significantly affect tumor cell proliferation and migration (**Supplementary Figures S3-S6**).

Taken together, these findings indicate that the factors play a pivotal role in affecting the proliferation and migration of ccRCC cells, further supporting its functional relevance and biological significance within the prognostic signature.

### 3.7 Knockdown of the signature factor FOXD2-AS1 reduces the expression of migrasome markers

**Figure 15:**
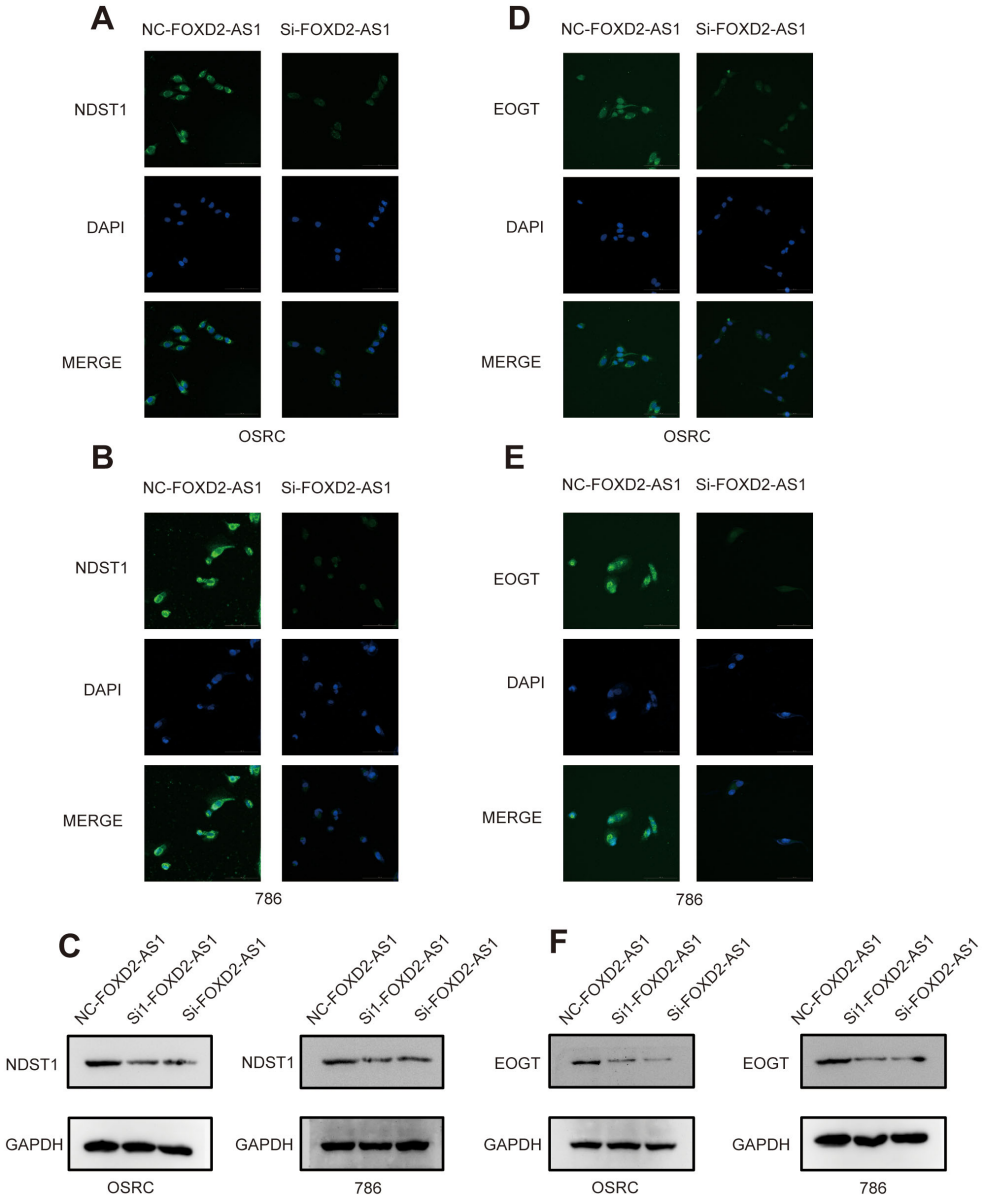
Knockdown of FOXD2-AS1 decreases the expression of migrasome markers. **(A, B)** Immunofluorescence assays show that the expression level of DNST1 is significantly decreased after knockdown of FOXD2-AS1. **(C)** Western blot analysis demonstrates that the protein expression level of DNST1 is reduced following FOXD2-AS1 knockdown. **(D, E)** Immunofluorescence assays show that the expression level of EOGT is significantly decreased after knockdown of FOXD2-AS1. **(F)** Western blot analysis demonstrates that the protein expression level of EOGT is reduced following FOXD2-AS1 knockdown.

We further sought to explore whether the factor in our signature directly affects the expression of migrasomes. To this end, we conducted relevant investigations based on the migrasome markers NDST1 and EOGT. After knocking down FOXD2-AS1, immunofluorescence assays showed a significant reduction in the fluorescence intensity of NDST1 (**Figures 15A, B**). Meanwhile, western blot experiments revealed that the expression level of NDST1 was indeed markedly decreased (**Figure 15C**). Consistent results were obtained when EOGT was detected after FOXD2-AS1 knock down (**Figures 15D-F**). These findings suggest that the factor in our signature can indeed influence migrasomes to a certain extent, thereby ultimately affecting tumorigenesis and development.

## 4 Discussion

In this study, we initially identified a set of migrasome-related genes, subsequently screening for lncRNAs associated with these genes using transcriptomic data from patients with ccRCC. These factors play diverse and critical roles in tumor biology. ITGB1 plays a role in promoting chordoma progression by binding to GMFG produced by ERS-CAFs, thereby enhancing tumor malignancy (16). ITGA5 is implicated in IGFBP2-mediated gefitinib resistance in NSCLC, serving as a critical player in this process (17). EOGT facilitates O-GlcNAcylation of NOTCH1 in pancreatic cancer, promoting the nuclear localization of the Notch intracellular domain (NICD) and this process contributes to the suppression of E-cadherin and P21 transcription, supporting PDAC progression (18). CPQ expression is elevated in glioblastoma tissues and inversely correlated with its methylation level. Low CPQ expression and high methylation are linked to better overall survival, indicating CPQ’s potential as a prognostic biomarker in glioblastoma (19). PIGK encodes a component of the GPI transamidase complex, essential for attaching GPI anchors to proteins. Variants in PIGK result in decreased cell surface levels of GPI-anchored proteins, leading to inherited GPI deficiency disorders (20). NDST1, an enzyme involved in heparan sulfate sulfation, plays a critical role in lymphatic metastasis by facilitating chemokine interactions in the lymphatic microenvironment (21). TSPAN4 serves as a migrasome marker in liver cancer, correlating with CD151 expression. Its association with CD151 suggests a role in promoting migrasome formation, contributing to liver cancer invasiveness and angiogenesis (22). PKD1 and PKD2 mutations cause autosomal dominant polycystic kidney disease (ADPKD), often leading to kidney failure. Variations in disease progression among patients suggest that factors beyond PKD1/2 mutations influence the rate of ADPKD advancement (23). ZNF503-AS1 acts as a tumor suppressor in bladder cancer by recruiting transcription factor GATA6 to up-regulate SLC8A1, which increases intracellular Ca2+ concentration. This process inhibits cell proliferation, invasion, and migration, while promoting apoptosis in bladder cancer cells (24). NARF-IT1, an m6A-related lncRNA, is identified as significantly associated with prognosis in myeloid leukemia (ML). Its expression, along with other lncRNAs like CRNDE and CHROMR, was confirmed in ML cell lines, suggesting potential for prognostic prediction in ML patients (25). FOXD2-AS1 promotes glioma progression by enhancing stemness and proliferation in glioma stem cells (GSCs) through the activation of the NOTCH signaling pathway via TAF-1 upregulation. Its silencing inhibits GSC stemness, reduces proliferation, and promotes apoptosis, highlighting its potential as a therapeutic target in glioma (26). In head and neck squamous cell carcinoma, AL031985.3 is an immune-related lncRNA linked to overall survival, suggesting its potential role as a prognostic marker and influence on the tumor microenvironment (27). LINC01843 is identified as a component of a pyroptosis-related five-lncRNA signature that correlates with prognosis and immune response in lung adenocarcinoma, showing potential relevance for predicting disease outcome and guiding immunotherapy strategies in cancer patients (28). GAS5-AS1 acts as a tumor suppressor in non-small cell lung cancer by inhibiting cell migration and invasion. Its reduced expression, often due to epigenetic silencing, is associated with enhanced epithelial-mesenchymal transition, promoting tumor metastasis (29). AL162377.1 is an upstream lncRNA in ccRCC that regulates SYDE2 expression through the miR-21-5p axis. This lncRNA-mediated pathway influences immune cell infiltration and has implications for ccRCC prognosis (30). Based on these lncRNAs, we developed a risk model for prognostic prediction. The model construction involved segregating patients into high- and low-risk groups to identify migrasome-associated lncRNAs significantly linked to prognosis, and its independent prognostic value was validated through multivariate Cox regression analysis.

To date, various prognostic signatures have been developed for ccRCC, and many have demonstrated utility in predicting patient outcomes. However, the predictive performance of these models varies depending on the factors they are based on. In this study, we compared our constructed signature with several previously published models. Notably, many existing signatures primarily report short-term predictive performance, whereas our model was evaluated using time-dependent AUCs at 1, 3, and 5 years. Importantly, our signature achieved a 5-year AUC of 0.758, which represents a remarkably high discriminative ability compared to other published models. This suggests that our model has strong potential for long-term prognostic prediction in ccRCC (31, 32). Moreover, unlike previous signatures, our signature is the first to be developed based on the migrasome, a novel and biologically significant organelle. This endows our signature with a cutting-edge biological foundation, enhancing not only its value in prognostic prediction but also its potential to provide insights into migrasome-related research.

To further confirm the signature’s robustness, we conducted multidimensional analyses of high- and low-risk groups, including assessments of the immune microenvironment, tumor mutation burden (TMB), and drug sensitivity, to explore the model’s applicability in prognostic evaluation for ccRCC. The tumor immune microenvironment plays a pivotal role in the dynamic and continuous interactions between the immune system and cancer cells, with immune evasion emerging as a central factor in cancer progression, from the initial development of cancer cells to the formation of metastatic disease (33). Immune checkpoint inhibition has emerged as an increasingly effective cancer immunotherapy. High TMB has been shown to predict clinical benefit from immune checkpoint inhibition across various cancer types. The TMB threshold associated with improved survival varies by cancer type, suggesting a strong correlation between elevated TMB and enhanced survival in patients receiving ICIs (34). Specifically, following model construction, we validated its efficacy through survival curve analysis. Results indicated that patients in the high-risk group exhibited significantly lower overall survival rates than those in the low-risk group, underscoring the potential of this migrasome-associated lncRNA signature for stratified prognostic prediction. Furthermore, immune function analysis highlighted substantial differences in immune cell infiltration and immune functions between high- and low-risk groups. The findings showed that patients in the high-risk group had a higher proportion of immunosuppressive cell types, such as regulatory T cells and M0 macrophages, aligning with the characteristics of TME. Tumor mutation burden (TMB) analysis revealed that the high-risk group exhibited significantly higher mutation frequencies compared to the low-risk group, particularly in key driver genes such as VHL and PBRM1. This finding aligns with the aggressive characteristics observed in high-risk patients. VHL missense mutations define an aggressive subtype of clear cell renal cell carcinoma (ccRCC) with poorer survival outcomes. ccRCC with VHL missense mutations exhibits distinct oncogenic features, including hyperactivation of cell cycle and NF-κB pathways, contributing to an inflamed tumor microenvironment (35). PBRM1 regulates PD-L1 expression by enhancing PBAF complex recruitment to the PD-L1 promoter. In clear cell renal cell carcinoma, alternative splicing of PBRM1 exon 27, mediated by RBFOX2, influences resistance to PD-1 blockade therapy (36). The marked TMB differences further suggest that migrasome-associated lncRNAs may influence mutation patterns in ccRCC and are potentially linked to the tumor’s immunological profile. Additionally, we conducted a drug sensitivity analysis to explore the model’s potential in guiding personalized therapy. The results showed that low-risk patients were more sensitive to drugs like Dihydrorotenone, OSI-027 and SB505124, whereas high-risk patients responded better to treatments such as Afuresertib, Entinostat and XAV939.

We also found that several results from our pathway enrichment analyses provide biologically plausible explanations for the differential drug sensitivity observed between the two risk groups. In the low-risk group, significant enrichment was observed in metabolic pathways, including oxidative phosphorylation, fatty acid metabolism, and PPAR signaling, indicating a greater dependency on metabolic processes. This may explain the increased sensitivity to metabolism-targeting agents in this subgroup. For instance, Dihydrorotenone, a mitochondrial complex I inhibitor, directly disrupts oxidative phosphorylation, which may underlie its enhanced efficacy in tumors highly reliant on mitochondrial energy metabolism, as seen in the low-risk group. OSI-027, a dual mTORC1/2 inhibitor, suppresses lipid biosynthesis and metabolism, thereby targeting PPAR-regulated lipid metabolic processes, which were also prominently enriched in the low-risk group (37, 38). Additionally, SB505124, a TGF-β type I receptor inhibitor, modulates TGF-β–mediated metabolic reprogramming and is relevant to pathways such as tyrosine metabolism, which were enriched in the low-risk group (39). In contrast, the high-risk group was predominantly enriched in immune-related pathways, including cytokine–cytokine receptor interaction, hematopoietic cell lineage, and extracellular matrix (ECM)–receptor interaction, suggesting a more active or dysregulated immune microenvironment. These features may account for the increased predicted sensitivity to drugs targeting immune modulation or associated signaling. For example, Afuresertib, an AKT inhibitor, interferes with downstream signaling of cytokine receptors, disrupting survival and immune evasion mechanisms in tumors (40). Entinostat, a histone deacetylase (HDAC) inhibitor, has been shown to enhance antigen presentation and modulate immune checkpoint expression, thereby promoting anti-tumor immunity—a mechanism consistent with the immune-related pathway enrichment in the high-risk group (41–43). Moreover, XAV939, a Wnt/β-catenin pathway inhibitor, can alleviate immunosuppression and disrupt ECM remodeling, aligning with the enrichment of ECM–receptor interaction and immune-related pathways in this group (44, 45). These findings support the biological relevance of our risk stratification and highlight the potential of the migrasome-related lncRNA signature to inform personalized therapeutic strategies based on pathway-specific drug vulnerabilities. These findings provide a potential reference for stratified treatment in ccRCC, suggesting that patients in specific risk groups may benefit from tailored therapeutic approaches. In addition, we integrated single-cell transcriptomic data to explore the underlying mechanisms by which the lncRNA-based prognostic signature may shape the tumor microenvironment in ccRCC. At single-cell resolution, we observed substantial differences in cellular composition between the high- and low-risk groups. Notably, high-risk tumors exhibited a greater abundance of tumor epithelial cells endothelial cells and plasma cells, suggesting enhanced tumor aggressiveness and abnormal angiogenic activity may contribute to the unfavorable prognosis.

These cell types also play an important role in the TME. Tumor epithelial cells secrete CCL9 and IL-23 in select tumors, establishing a pro-inflammatory, pro-angiogenic, and immunosuppressive tumor microenvironment that significantly accelerates tumor progression (46). ​Tumor endothelial cells play pivotal roles not only in supporting tumor growth but also in actively orchestrating immune evasion. Particularly, a specialized subset termed immunomodulatory endothelial cells (IMECs) exhibits remarkable plasticity to dynamically regulate immune responses during tumor progression (47). The IgG-FcγRIIA axis orchestrates plasma cell-mediated maintenance of glioblastoma stem cell stemness in glioblastoma multiforme, propelling tumorigenic programs through enhanced self-renewal and proliferative capacity (48).

In our previous survival analysis of bulk RNA seq, we found that the high-risk group had a poorer survival prognosis. Among the cell types rated as high-risk in these single-cell analyses, we discovered tumor epithelial cells had relatively significant cell communication with T cells, and at the same time, endothelial cells also had relatively significant cell communication with tumor epithelial cells. Tumor epithelial cells actively secrete VEGF, which stimulates endothelial cell proliferation and triggers pathological angiogenesis through multifaceted signaling cascades, ultimately fostering tumor neovascularization. VEGF induces upregulation of the Notch ligand DLL4 in endothelial cells via VEGFR2 activation, whereby DLL4-mediated Notch signaling differentially suppresses VEGFR2/3 expression in adjacent cells to spatially restrict endothelial sprouting and proliferation—preserving VEGFR2-dependent angiogenic competence while restraining excessive branching through Notch-imposed inhibition of VEGFR3 (49).

Moreover, several key lncRNAs within the signature exhibited distinct cell-type–specific expression patterns, indicating their potential involvement in endothelial activation, epithelial transformation, and immune modulation, respectively. Together, these single-cell analyses not only provide biological validation of the prognostic model, but also uncover critical tumor–immune–stromal interactions that may underlie risk stratification. These findings lay the groundwork for future mechanistic investigations and may inform the development of cell type–specific therapeutic strategies for ccRCC. We experimentally validated the oncogenic role of FOXD2-AS1, a key component of our migrasome-associated lncRNA signature, in promoting ccRCC progression. Knockdown of FOXD2-AS1 significantly reduced cell migration and proliferation in ccRCC cells, confirming its biological relevance. Although FOXD2-AS1 has a lower coefficient in the signature, its experimental accessibility and stable expression in ccRCC made it an ideal candidate for validation. These findings further support the predictive robustness of the lncRNA signature and highlight FOXD2-AS1 as a potential therapeutic target.

In summary, this study demonstrates that migrasome-associated lncRNAs hold significant clinical potential in prognostic prediction, immune modulation, and drug sensitivity for clear cell renal cell carcinoma (ccRCC). One limitation of this study is the lack of external validation due to limited data availability, which we aim to address in future research as more independent datasets become available. Although the signature developed in this study demonstrated promising prognostic value, its clinical implementation requires further exploration. With the increasing accessibility and cost-effectiveness of transcriptome-based technologies such as qRT-PCR and RNA-seq, the detection of lncRNA expression in biopsy samples is becoming more feasible in clinical practice. Furthermore, considering the anatomical characteristics of renal cell carcinoma, we propose that future studies investigate the potential of detecting signature-related lncRNAs in non-invasive specimens such as urine. This approach, although still preliminary, may provide a novel direction for developing non-invasive prognostic tools. Nevertheless, additional validation in large-scale, multi-center cohorts will be essential before this signature can be translated into routine clinical application. Future research should further investigate the molecular mechanisms of these lncRNAs in renal cancer to enhance the clinical translational applications of the signature.

## 5 Conclusions

This study proposes a novel prognostic signature based on migrasome-associated long non-coding RNAs (lncRNAs) and validates its significant prognostic potential in clear cell renal cell carcinoma (ccRCC). By integrating transcriptomic and clinical data, we identified lncRNAs closely associated with migrasome-related genes and constructed a robust risk prediction model. This signature effectively stratifies ccRCC patients into distinct risk groups with significant survival differences and uncovers critical associations with immune cell infiltration, tumor mutation burden, and drug sensitivity. Furthermore, single-cell transcriptomic analysis revealed cell type–specific expression patterns of the signature genes and distinct microenvironmental features across risk groups, reinforcing the model’s biological interpretability. The findings highlight the potential clinical utility of this model in guiding personalized treatment for ccRCC and emphasize the practical value of migrasome-associated lncRNAs, offering new insights for further exploration of their biological functions and therapeutic applications.

## Data Availability

The original contributions presented in the study are included in the article/**Supplementary Material**. Further inquiries can be directed to the corresponding authors.
