# Supplementary material for: Integrative bulk and single-cell transcriptomic analysis identifies a migrasome-associated lncRNA signature predictive of prognosis and immune landscape in clear cell renal cell carcinoma
Source: Front Immunol. 2025 Aug 20;16:1638792. doi: 10.3389/fimmu.2025.1638792 (PMC12404980; doi:10.3389/fimmu.2025.1638792)
Supplement: Supplementary file 1 [file DataSheet1.pdf]

Supplementary Material

1 Supplementary Figures

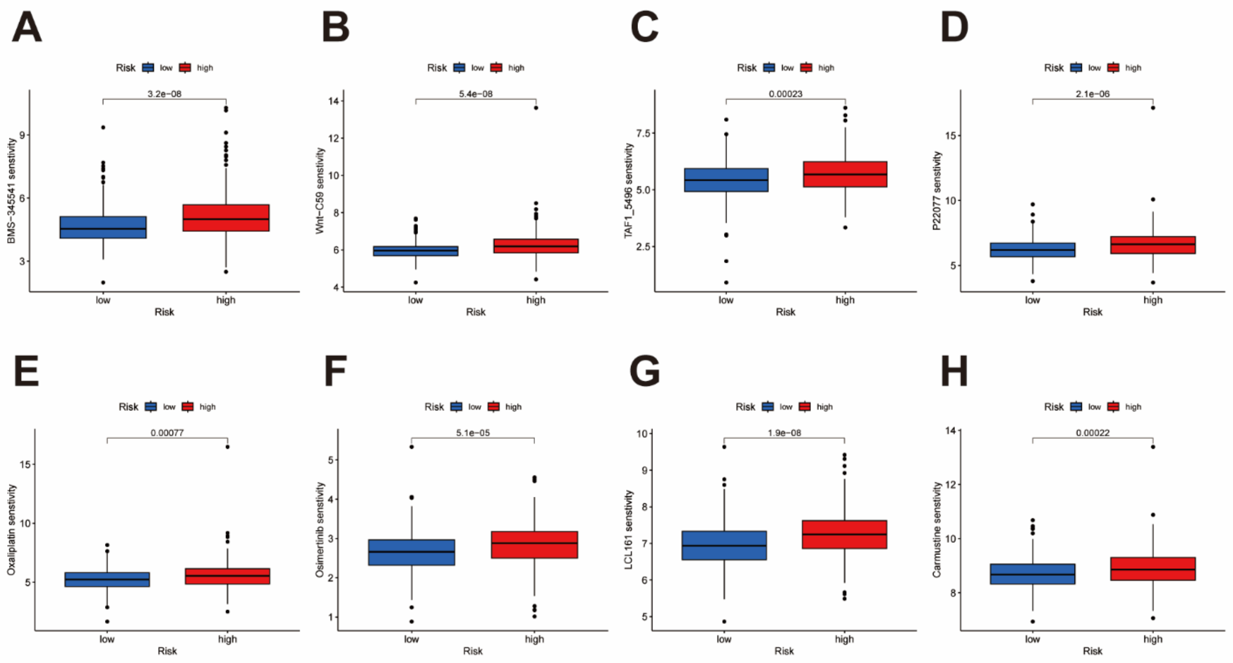

Supplementary Figure S1 (A-H) Drug sensitivity analysis revealed the following drugs to be predicted as more sensitive in the low-risk group.

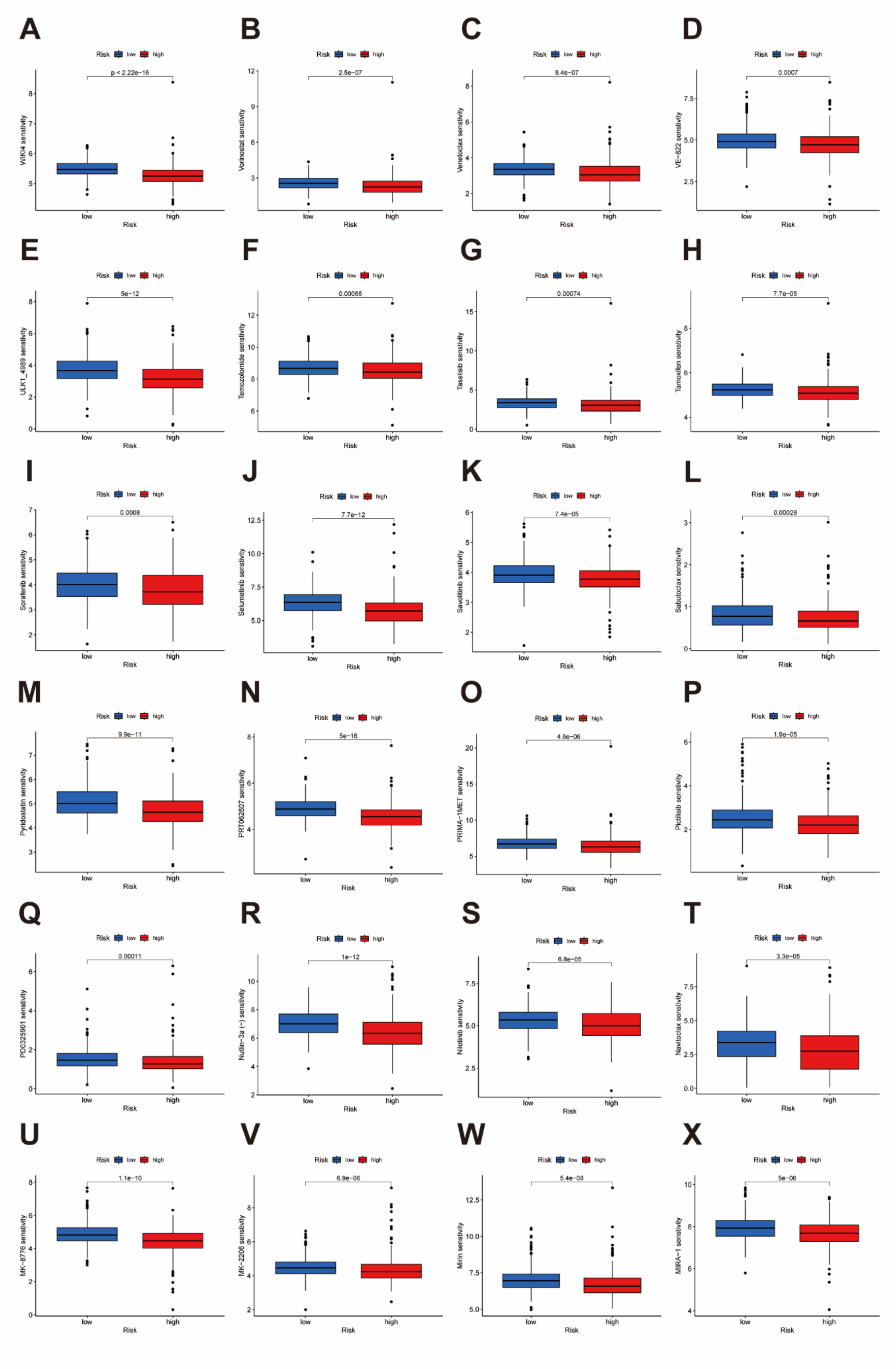
A
Risk low high
p < 2.22e−16
WIKI4 sensitivity
low high
Risk
B
Risk low high
2.5e−07
Vorinostat sensitivity
low high
Risk
C
Risk low high
8.4e−07
Venetoclax sensitivity
low high
Risk
D
Risk low high
0.0007
VE−822 sensitivity
low high
Risk
E
Risk low high
5e−12
ULK1_4989 sensitivity
low high
Risk
F
Risk low high
0.00065
Temozolomide sensitivity
low high
Risk
G
Risk low high
0.00074
Taselisib sensitivity
low high
Risk
H
Risk low high
7.7e−05
Tamoxifen sensitivity
low high
Risk
I
Risk low high
0.0008
Sorafenib sensitivity
low high
Risk
J
Risk low high
7.7e−12
Selumetinib sensitivity
low high
Risk
K
Risk low high
7.4e−05
Savolitinib sensitivity
low high
Risk
L
Risk low high
0.00028
Sabutoclax sensitivity
low high
Risk
M
Risk low high
9.9e−11
Pyridostatin sensitivity
low high
Risk
N
Risk low high
5e−16
PRT062607 sensitivity
low high
Risk
O
Risk low high
4.6e−06
PRIMA−1MET sensitivity
low high
Risk
P
Risk low high
1.9e−05
Pictilisib sensitivity
low high
Risk
Q
Risk low high
0.00011
PD0325901 sensitivity
low high
Risk
R
Risk low high
1e−12
Nutlin−3a (−) sensitivity
low high
Risk
S
Risk low high
6.8e−05
Nilotinib sensitivity
low high
Risk
T
Risk low high
3.3e−05
Navitoclax sensitivity
low high
Risk
U
Risk low high
1.1e−10
MK−8776 sensitivity
low high
Risk
V
Risk low high
6.9e−06
MK−2206 sensitivity
low high
Risk
W
Risk low high
5.4e−08
Mirin sensitivity
low high
Risk
X
Risk low high
5e−06
MIRA−1 sensitivity
low high
Risk

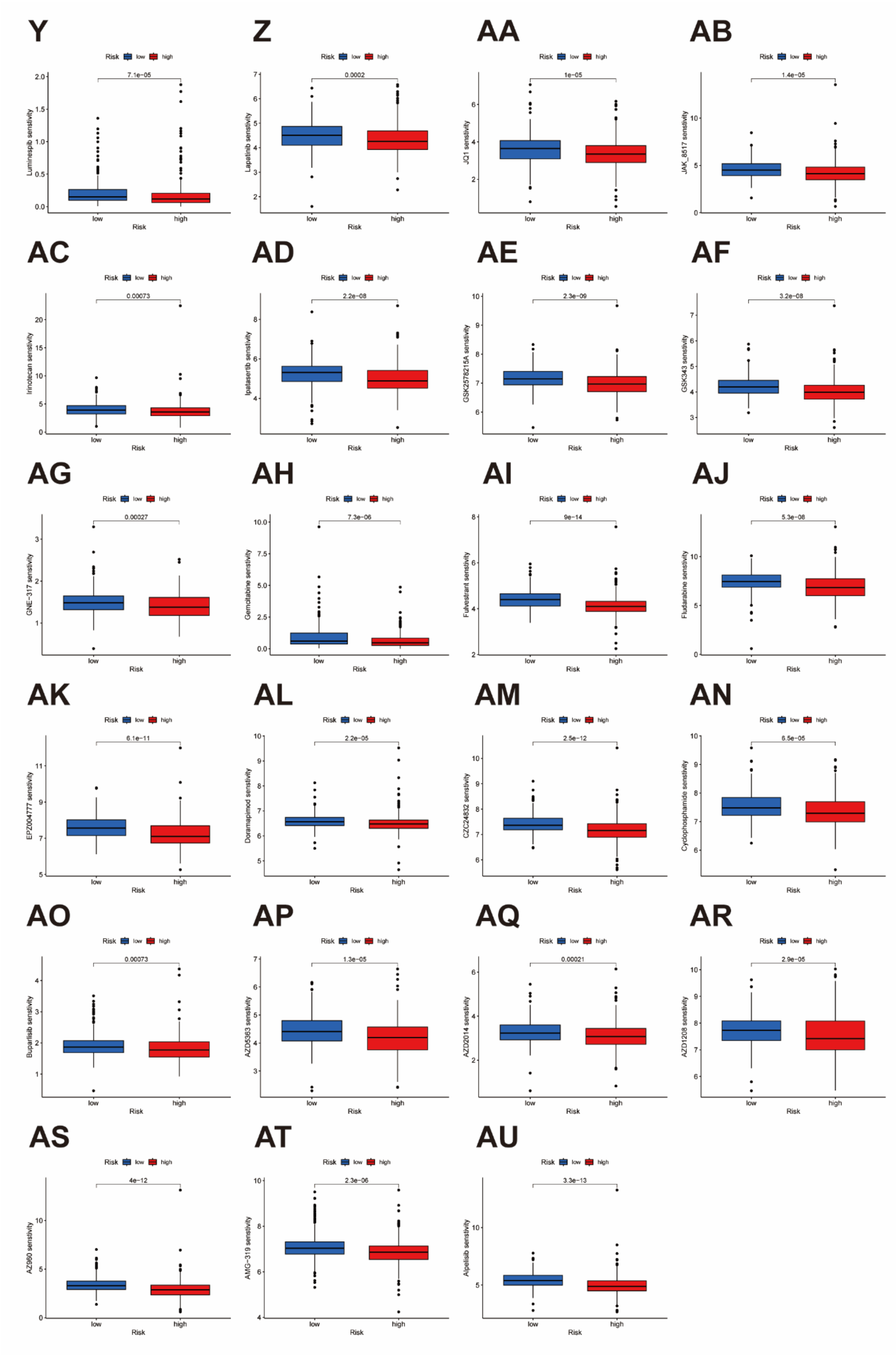

Supplementary Figure S2 (A-AU) Drug sensitivity analysis revealed the following drugs to be predicted as more sensitive in the high-risk group.

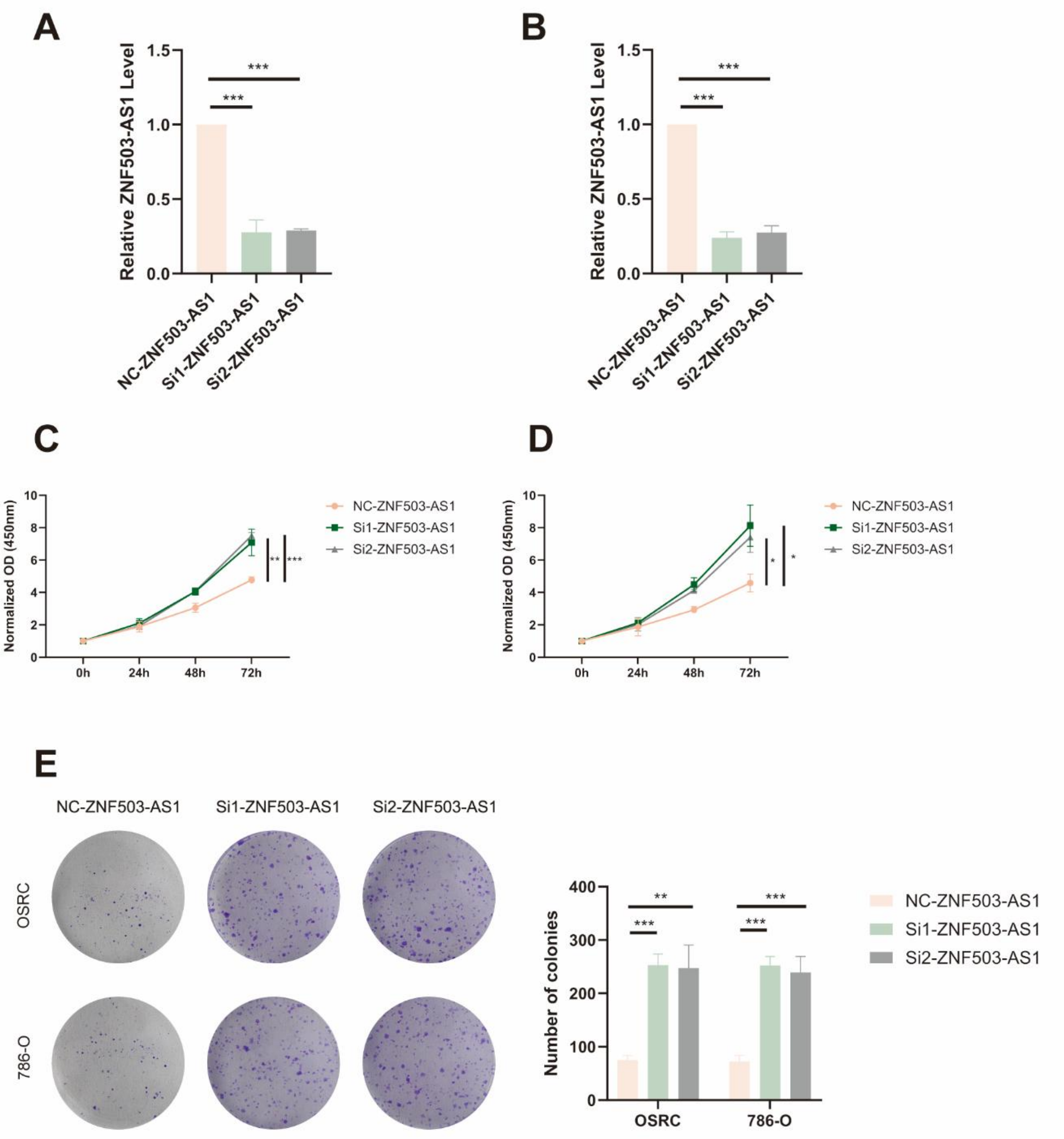

Supplementary Figure S3 (**A-B**) qRT-PCR analysis showing the knockdown efficiency of ZNF503-AS1 in OS-RC-2 and 786-O cells after siRNA transfection. (**C, D**) CCK-8 assays demonstrating that silencing ZNF503-AS1 significantly enhances the proliferation of OS-RC-2 and 786-O cells over time. (**E**) Colony formation assay showing a marked increase in the number and size of colonies formed by ZNF503-AS1-silenced cells compared to controls.

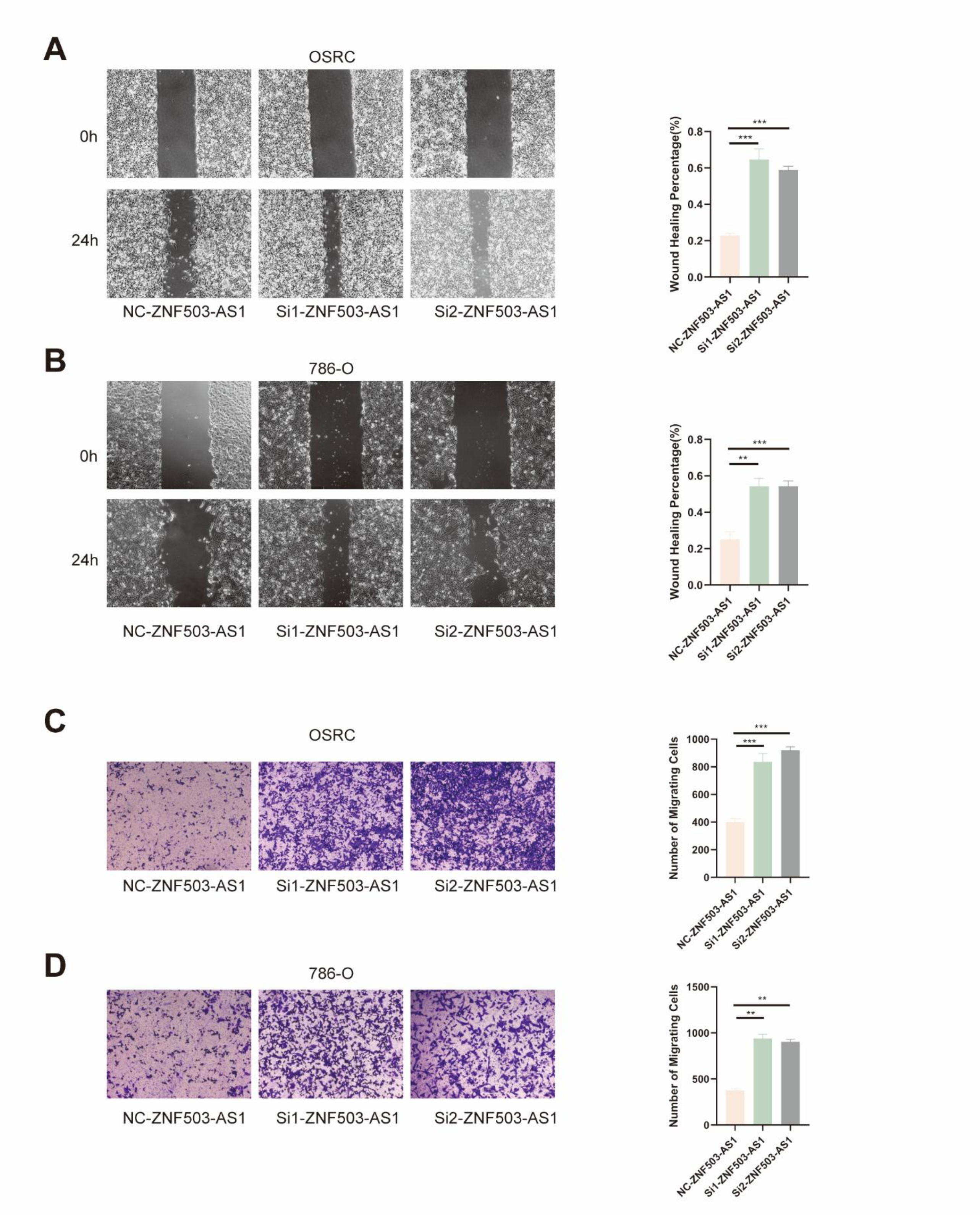

Supplementary Figure S4 (**A, B**) Wound healing assay showing that ZNF503-AS1 knockdown enhances cell migration, as indicated by increased wound closure after 24 hours. (**C, D**) Transwell migration assay further confirming that ZNF503-AS1 silencing significantly increases the number of migrating OS-RC-2 and 786-O cells.

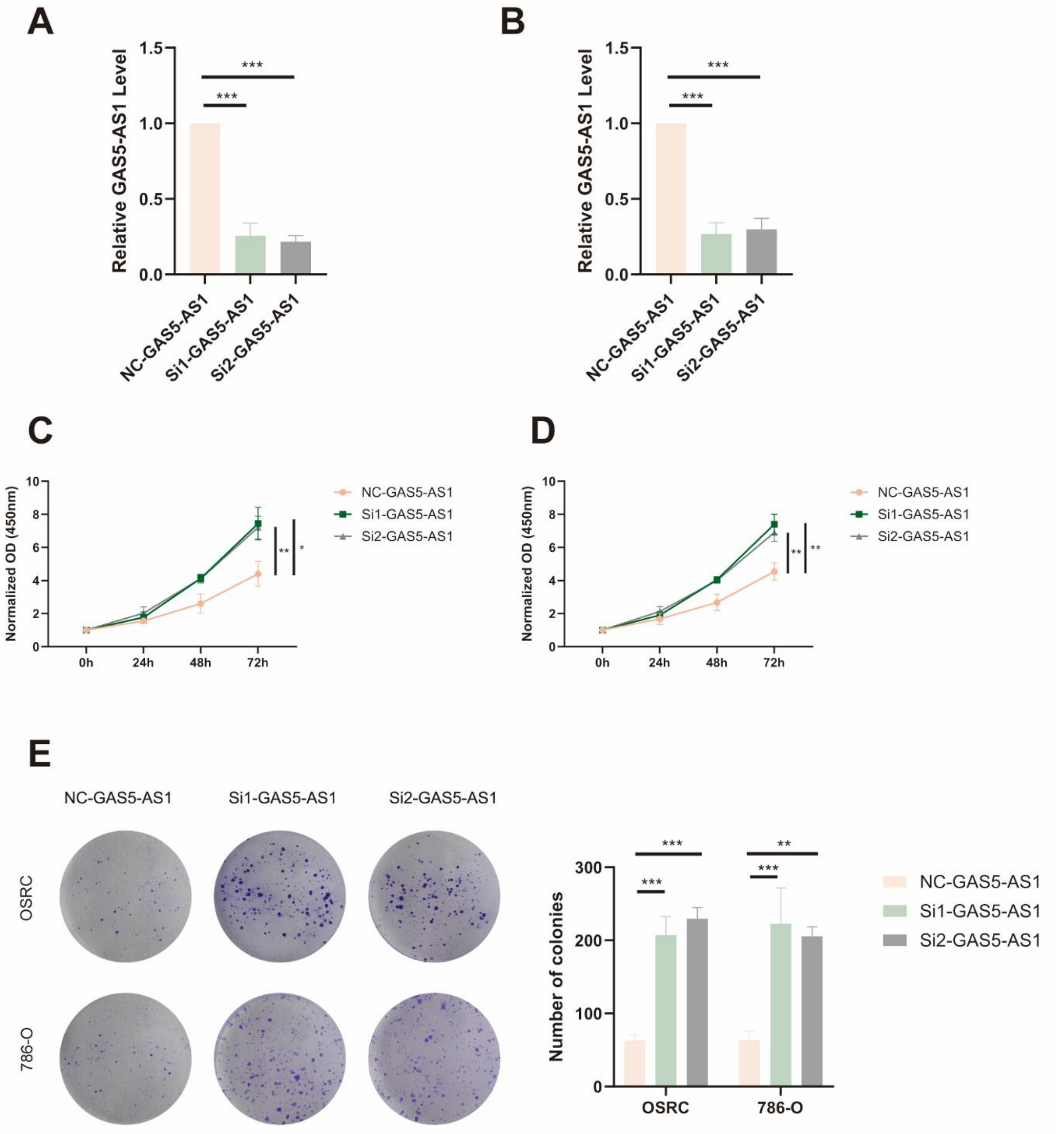

Supplementary Figure S5 (**A-B**) qRT-PCR analysis showing the knockdown efficiency of GAS5-AS1 in OS-RC-2 and 786-O cells after siRNA transfection. (**C, D**) CCK-8 assays demonstrating that silencing GAS5-AS1 significantly enhances the proliferation of OS-RC-2 and 786-O cells over time. (**E**) Colony formation assay showing a marked increase in the number and size of colonies formed by GAS5-AS1-silenced cells compared to controls.

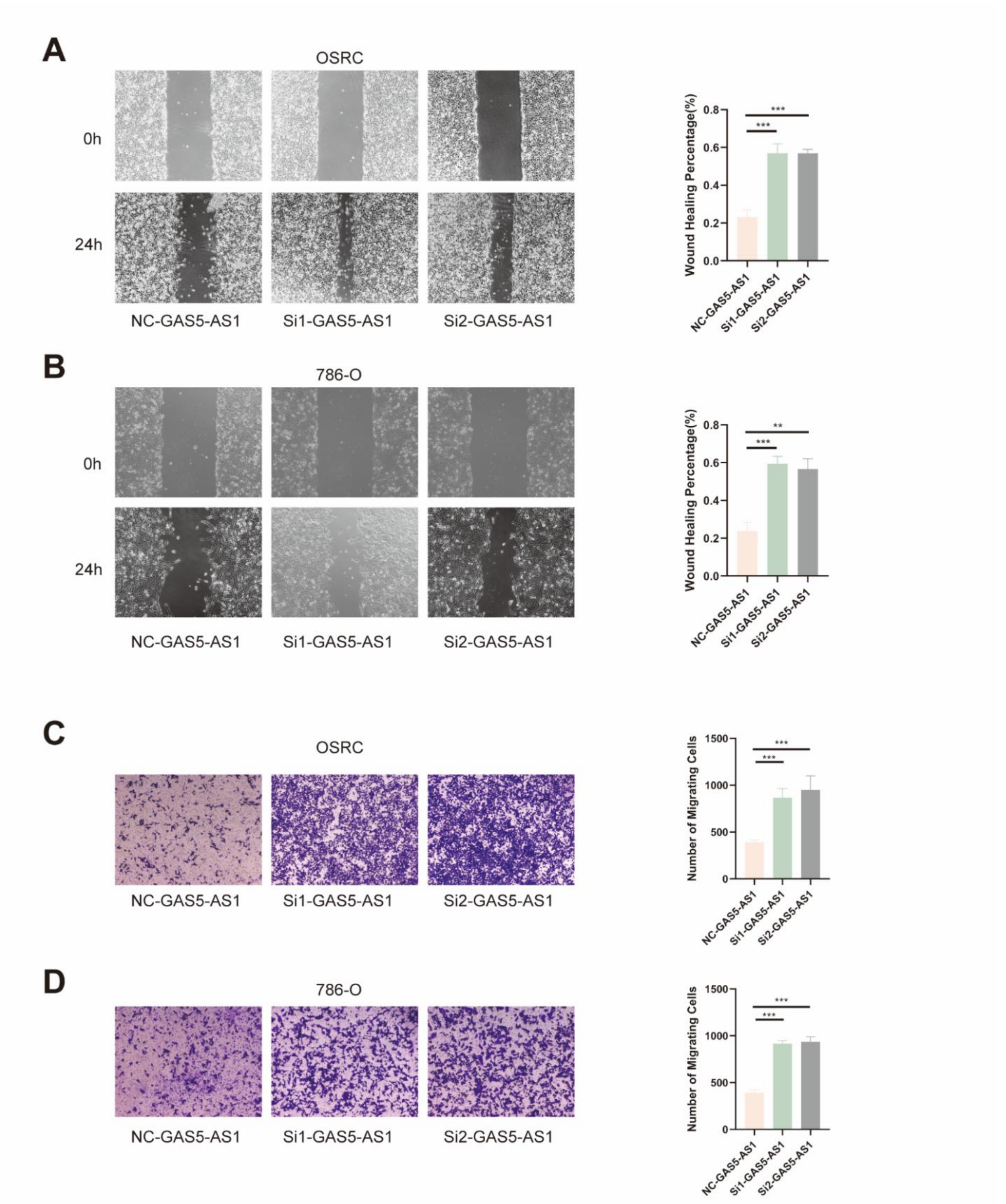

Supplementary Figure S6 (**A, B**) Wound healing assay showing that GAS5-AS1 knockdown enhances cell migration, as indicated by increased wound closure after 24 hours. (**C, D**) Transwell migration assay further confirming that GAS5-AS1 silencing significantly increases the number of migrating OS-RC-2 and 786-O cells.

2 Supplementary Tables

	Primers sequence (5′-3′)
FOXD2-AS1-F	5′-GCCCAGAACAATTGGGAGGA-3′
FOXD2-AS1-R	5′-AAGAGAGGGAGAGACGACCC-3′
ZNF503-AS1-F	5′-TAACTGGTGAAGCCCGGAAG-3′
ZNF503-AS1-R	5′-TCGACTGGGTCATTAAATGCT-3′
GAS5-AS1-F	5′-TCCCAGCCTCAGACTCAACA-3′
GAS5-AS1-R	5′-GTTTCATAGGCCCCTGTGCT-3′
GAPDH-F	5′- ACAACTTTGGTATCGTGGAAGG-3′
GAPDH-R	5′- GCCATCACGCCACAGTTTC-3′
si FOXD2-AS1#1 Sense strand	5′-GCGAAGAGUACGUUGCUAUTT-3′
si FOXD2-AS1#1 Antisense strand	5′- AUAGCAACGUACUCUUCGCTT -3′
si FOXD2-AS1#2 Sense strand	5′- GUUCGAGAGUGAAUUUACATT -3′
si FOXD2-AS1#2 Antisense strand	5′- UGUAAAUUCACUCUCGAACTT -3′

si ZNF503-AS1#1 Sense strand	5′- GGAAGAGCUUGUUGUUCAAGATT-3′
si ZNF503-AS1#1 Antisense strand	5′- UCUUGAACAACAAGCUCUUCCTT -3′
si ZNF503-AS1#2 Sense strand	5′- CAGACUAGUUGAAGGUAGAGATT -3′
si ZNF503-AS1#2 Antisense strand	5′- GGAAGAGCUUGUUGUUCAAGATT -3′
si GAS5 -AS1#1 Sense strand	5′- GCUAACAAAGACAUCAUUAUATT-3′
si GAS5 -AS1#1 Antisense strand	5′- UAUAAUGAUGUCUUUGUUAGCTT -3′
si GAS5 -AS1#2 Sense strand	5′- CCAAGUAGCUCCAUCAAUACUTT -3′
si GAS5-AS1#2 Antisense strand	5′- AGUAUUGAUGGAGCUACUUGGTT -3′

Supplementary Table 1 The sequences of qRT-PCR primers and siRNA

Covariates	Type	Total	Test	Train	Pvalue
**Age**	<=65	349(65.48%)	179(67.29%)	170(63.67%)	0.4304
	>65	184(34.52%)	87(32.71%)	97(36.33%)	
**Gender**	FEMALE	188(35.27%)	93(34.96%)	95(35.58%)	0.9532
	MALE	345(64.73%)	173(65.04%)	172(64.42%)	
**Grade**	G1	14(2.63%)	9(3.38%)	5(1.87%)	0.3939
	G2	229(42.96%)	107(40.23%)	122(45.69%)	
	G3	206(38.65%)	104(39.1%)	102(38.2%)	
	G4	76(14.26%)	42(15.79%)	34(12.73%)	
	unknow	8(1.5%)	4(1.5%)	4(1.5%)	
**Stage**	Stage I	267(50.09%)	124(46.62%)	143(53.56%)	0.1676
	Stage II	57(10.69%)	27(10.15%)	30(11.24%)	
	Stage III	123(23.08%)	63(23.68%)	60(22.47%)	
	Stage IV	83(15.57%)	50(18.8%)	33(12.36%)	
	unknow	3(0.56%)	2(0.75%)	1(0.37%)	
**T**	T1	273(51.22%)	127(47.74%)	146(54.68%)	0.1954
	T2	69(12.95%)	34(12.78%)	35(13.11%)	
	T3	180(33.77%)	97(36.47%)	83(31.09%)	
	T4	11(2.06%)	8(3.01%)	3(1.12%)	
**M**	M0	422(79.17%)	203(76.32%)	219(82.02%)	0.0521
	M1	79(14.82%)	48(18.05%)	31(11.61%)	
	unknow	32(6%)	15(5.64%)	17(6.37%)	
**N**	N0	240(45.03%)	122(45.86%)	118(44.19%)	0.5184
	N1	16(3%)	10(3.76%)	6(2.25%)	
	unknow	277(51.97%)	134(50.38%)	143(53.56%)	

Supplementary Table 2 Clinical characteristics between groups and p-values for differences between the training and testing sets.
